# Electromagnetic Radiation Stimulated Learning in Perovskite Nickelates

**DOI:** 10.1002/advs.75984

**Published:** 2026-06-12

**Authors:** Ranjan Kumar Patel, Kabir Zama, Matthew Smart, Ramya Eathirajan, Ivan Seskar, Narayan Mandayam, Guangxin Ni, Martin Mönnigmann, Shriram Ramanathan

**Affiliations:** ^1^ Department of Electrical and Computer Engineering Rutgers University Piscataway New Jersey USA; ^2^ Lewis‐Sigler Institute of Integrative Genomics Princeton University Princeton New Jersey USA; ^3^ Department of Physics Florida State University Tallahassee Florida USA; ^4^ National High Magnetic Field Laboratory Tallahassee Florida USA; ^5^ WINLAB Rutgers University North Brunswick New Jersey USA; ^6^ Department of Mechanical Engineering Ruhr University Bochum, Bochum, Germany

**Keywords:** electromagnetic radiation, glassy dynamics, habituation, multi‐timescale memory, neuromorphic materials, perovskite nickelates, synaptic plasticity

## Abstract

Biological plasticity refers to the ability of synapses to strengthen or weaken over time. These adaptive properties play a fundamental role in learning and memory, spanning many orders of magnitude in timescales. Short‐term plasticity (STP) arises from rapid correlative activity, while long‐term plasticity (LTP) is governed by slower biochemical processes. Here, we investigate electromagnetically driven relaxation dynamics in perovskite nickelate thin films as an analogue of biological learning behaviors. By comparing radio frequency (RF), infrared (IR), visible, and ultraviolet (UV) radiation as stimuli, we find that RF excitation primarily induces STP, while visible and IR illumination lead to reversible relaxation on behavioral timescales. In contrast, UV illumination results in persistent, non‐thermal changes in conductivity over extended timescales. Notably, UV‐exposed nickelate films exhibit glass‐like dynamics, characterized by stretched exponential relaxation and aging phenomena. The films display habituation to repeated stimuli, along with sensitization and spontaneous recovery under controlled environments. A minimal dynamical systems model captures key qualitative features of the UV‐induced resistance changes. Our results demonstrate that electromagnetic frequency enables multi‐timescale relaxation spanning nearly nine orders of magnitude, suggesting perovskite nickelates as promising platforms for adaptive optoelectronic hardware and for linking computational neuroscience with emerging quantum technologies.

## Graphical abstract

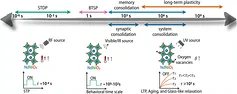

## 1 Introduction

**FIGURE 1:**
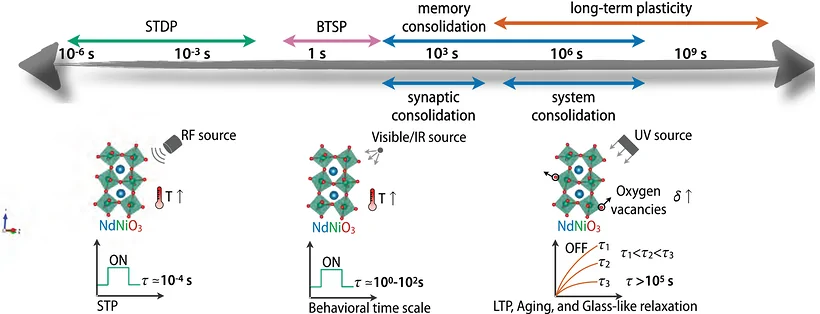
Schematic representation of multi‐timescale memory processes and stimulus‐driven plasticity in nickelate thin films. Synaptic and cellular memory mechanisms, such as spike‐timing dependent plasticity (STDP) and behavioral time‐scale plasticity (BTSP), operate over tens of milliseconds and seconds, respectively, enabling faster changes in synaptic strength or neural activity. Memory consolidation reorganizes activity and connectivity across larger brain areas and is primarily of two types: synaptic consolidation (minutes to hours) and systems consolidation (days to years). Additionally, long‐term plasticity (LTP) is governed by molecular and genetic processes that function over hours to years. Frequency‐dependent plasticity in nickelate thin films mimics these biological memory processes across multiple timescales: RF induces short‐term plasticity (STP) via thermal effects, visible and infrared illumination induce relaxation on behavioral timescales, while UV exposure triggers persistent, non‐thermal transformations through oxygen vacancy (δ) formation, resembling LTP and exhibiting glass‐like relaxation behavior with aging phenomena.

Synaptic plasticity in animal brains occurs over a wide range of timescales, from milliseconds to hours/days. The short‐term plasticity (STP) is often related to Hebbian learning and correlative effects, while longer time scale changes are often ascribed to slower learning and memory consolidation processes (see Figure 1 for different time scales). In neuroscience, plasticity is naturally separated into multiple phases, namely induction, expression, and consolidation [1]. The slow and nonlinear changes that extend into the seconds and longer timescales are of increasing interest in the context of behavioral learning and homeostasis in neural circuits. In the brain, these long‐time scale dynamics are governed by biochemical pathways such as availability of calcium or other ions, enzyme activity, neurotransmitter release kinetics, or sub‐threshold amplitude of action potential [2]. Neuromorphic hardware aims to emulate biological neural elements and therefore requires systems that can relax over a range of timescales, which contribute to implementing learning algorithms at the chip level. When one thinks of slow relaxations, arguably the most relevant material classes that come to mind are glasses. Glassy materials are well‐known for aging and dynamic phenomena that can extend over multiple frequencies [3]. In the context of neuromorphic computing, it is therefore worth investigating glasses whose functional properties such as electrical resistance can evolve over time either in the presence of or post‐exposure to stimuli. When the external stimulus is light, then it becomes very interesting not only for opto‐electronics, but also for the emerging field of machine vision [4]. Examples include autonomous vehicles that use optical sensors for self‐navigation as well as haptic interfaces in robotics. For instance, a photoresistor can be used to modify the output characteristics of an oscillator circuit as a means of sensing radiation [5]. More direct applications would simply utilize light as a stimulus for non‐contact modification of short or long‐term synaptic weights in a network [6]. The latter is particularly interesting in the context of post‐fabrication modification of electrical properties of neuromorphic hardware.

In this manuscript, we focus on the response of perovskite nickelates to RF, IR, visible, and UV radiation, respectively. Perovskite nickelates (e.g., ${\mathrm{NdNiO}}_{3}$, ${\mathrm{SmNiO}}_{3}$, etc.) are a class of crystalline quantum materials that show highly nonlinear electrical properties with carrier doping due to strong dependence on orbital filling. The strong Coulomb interactions inherently present in these system, along with their fragile band structures, offer a route for glassy dynamics to be present even in single crystals of nickelates. Typically, slow relaxations in such systems have been observed proximal to structural or electronic phase transition boundaries [7, 8, 9]. This manifests as a dynamic change in electrical resistance with time extending over 1000s of seconds when the material is positioned either close to or right in the middle of an insulator‐to‐metal transition at a particular temperature.

Besides the proximity to transition temperature as a resistance tuning knob, electron donors such as oxygen vacancies or hydrogen can significantly increase the electrical resistance due to half‐filling of the $e_{g}$ orbital. While changes to electrical conductivity due to carrier injection from dopants have been studied to some extent in literature, interaction of microwave or optical radiation with the nickelate systems is in early stages [10, 11, 12, 13, 14]. Here, we investigate the interaction of microwave radiation [2.4 GHz (9.93 μeV)], IR radiation [3.3 μm (0.38 eV)], visible light [red, 650 nm (1.91 eV)], and UV radiation [254 nm (4.88 eV) and 365 nm (3.4 eV)] with three prototypical nickelate systems: ${\mathrm{NdNiO}}_{3}$ (NNO), ${\mathrm{SmNiO}}_{3}$ (SNO), and H‐${\mathrm{NdNiO}}_{3}$ (HNNO) thin films. The pristine NNO is a metal, and the pristine SNO is a narrow gap semiconductor at room temperature with a bandgap of ∼0.5 eV [15], while hydrogen‐doped NNO is an insulator with a bandgap of ∼3 eV [16]. It is therefore an interesting comparison to understand photon energy effects on tuning electrical conductivity as well as investigate persistent changes that exist once the light is turned OFF. As we will discuss later, in complex oxides, it has been reported that persistent photoconductivity (PPC) is prevalent and has been associated with defects and long‐lived states with slow recombination dynamics [17, 18, 19, 20]. Our work presents an in‐depth study of photon effects on temporal changes to electrical conductivity, discerning thermal versus non‐thermal effects as well as a demonstration of non‐associative neuromorphic learning mechanisms in a non‐contact mode. Our results establish that UV light can effectively serve as a mechanism to induce glassy behavior in the nickelates at room temperature. To provide a quantitative framework for these multi‐timescale dynamics, we develop minimal dynamical systems models using memory variables to account for different timescales of material response to optical stimulation. These models, detailed in the Supporting Information, capture key qualitative features of the experimental observations and provide insights into the underlying temporal mechanisms.

## 2 Results and Discussion

### 2.1 Structural and Electrical Characterization

**FIGURE 2:**
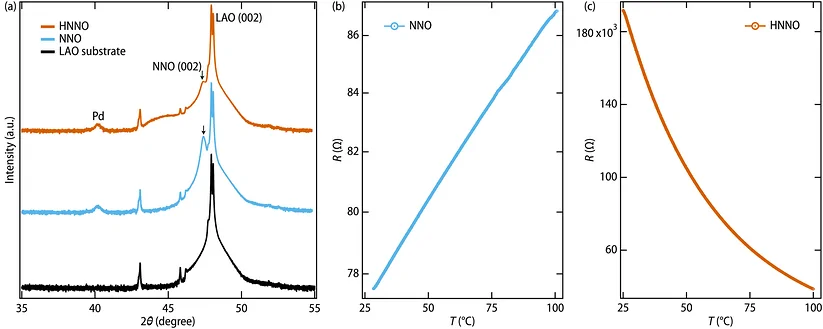
Structural and electrical characterizations of the NNO and HNNO films. (a) 2θ‐ω XRD scan of the NNO and HNNO films on LAO substrates, along with a reference spectrum for the LAO substrate. The NNO film peak is marked with (↓) symbol. Data have been vertically offset along the intensity axis for clarity. (b,c) Temperature‐dependent resistance of the NNO and HNNO films, respectively. NNO exhibits metallic behavior, whereas HNNO remains insulating over the measured temperature range from room temperature to 100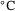 with three orders of magnitude change in resistance compared to pristine NNO at room temperature.

A ∼50 nm thick NNO film was deposited on a single‐crystalline (100)‐oriented LAO substrate using an RF sputtering system. The HNNO film was formed by annealing the NNO film in a forming gas environment at $150^{\circ }C$. Figure 2a shows the 2θ‐ω x‐ray diffraction (XRD) scan of the NNO and HNNO films, along with a reference scan of a bare LAO substrate around the (002)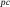 peak (where pc denotes the pseudo‐cubic notation). The XRD patterns of both NNO and HNNO films on the LAO substrate exhibit substrate peaks and a film peak (marked by ↓), confirming good structural quality of the films. The out of plane lattice constant ($c_{\mathrm{pc}}$) of the NNO film is calculated as 3.83 Å, consistent with literature [21, 22]. In the HNNO film, the XRD peak of the NNO film shifts slightly toward a lower angle, indicating an increase in hydrogen concentration with H‐doping [23]. Following the structural characterization, we investigated the electrical transport properties of the films. Bulk NNO exhibits an orthorhombic metallic phase at room temperature and undergoes an MIT around 200 K accompanied by a structural transition to a monoclinic phase [24, 25, 26]. In contrast, hydrogen doping is known to induce insulating behavior in HNNO films [23]. Figure 2b and Figure 2c show the transport characteristics over a temperature range from room temperature to 100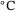 of NNO and HNNO films, respectively. While NNO film maintains its metallic nature with a constant slope of 0.13 Ω/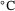, HNNO film exhibits insulating behavior within this temperature range. Notably, the room‐temperature resistance of the NNO film increases by three orders of magnitude after forming gas annealing, indicating successful hydrogen doping into the NNO lattice [16, 21, 23].

### 2.2 RF‐Driven Short‐Term Plasticity Emulation

**FIGURE 3:**
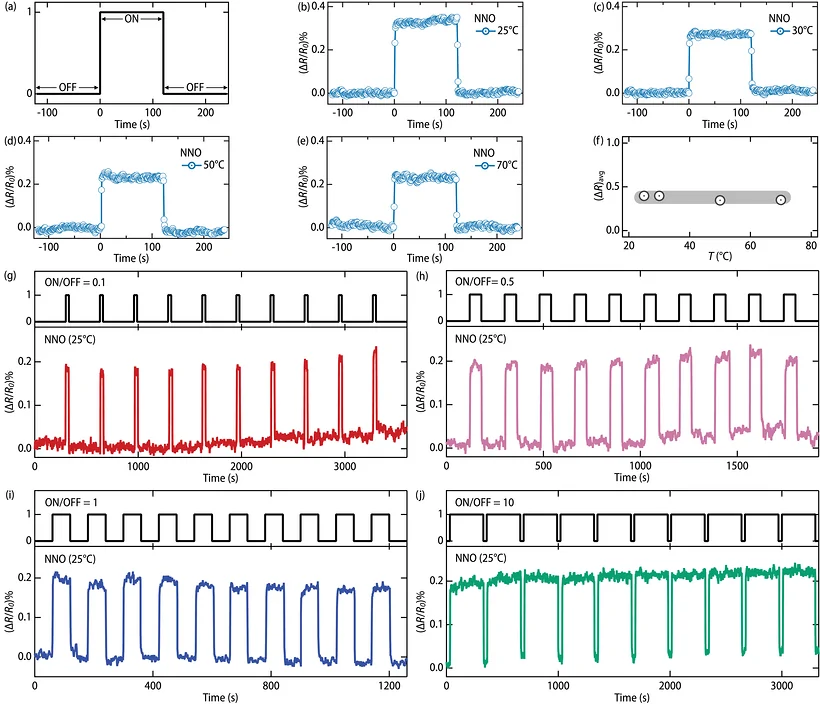
Interaction of RF radiation with NNO film at different temperatures and reversible resistance change with RF radiation. (a) A schematic illustration of the time‐dependent ON/OFF state of the RF signal, where the ON state is maintained for 2 min. (b–e) RPCR of NNO film under RF radiation of 2.4 GHz at $25^{\circ }C$, $30^{\circ }C$, $50^{\circ }C$, and $70^{\circ }C$, respectively. A sharp increase (decrease) of resistance is observed when the RF signal is turned ON (OFF). (f) The average change in resistance during the ON state as a function of temperature. A constant (${\Delta R)}_{avg}$ suggests a temperature effect induced by RF radiation. (g–j) RPCR of the NNO film at $25^{\circ }C$ with ON/OFF ratios of RF radiation set to 0.1, 0.5, 1, and 10, respectively, showing reversible resistance change with RF signal. Schematic representations of the RF ON/OFF state is shown in the upper panels of (g–j).

Following the investigation of the structural and electrical properties of the films, we examined the response of these films to RF radiation exposure. Figure 3a shows a schematic representation of ON/OFF states of the RF signal, where the RF radiation was ON for 2 min (same ON/OFF duration has been maintained for the measurements in Figure 3b–e). The pristine NNO sample was first heated to the desired temperature and stabilized for 15 min. Following this, the NNO film was exposed to RF radiation for 2 min while its resistance was monitored. Figures 3b–3e illustrate the relative percentage change in resistance (RPCR) as a function of time at $25^{\circ }C$, $30^{\circ }C$, $50^{\circ }C$, and $70^{\circ }C$, respectively. The RPCR is calculated as [(*R*‐*R* 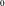)/*R* 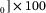, where *R* 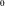 represents the initial resistance before turning ON the RF signal. The NNO film at all measured temperature exhibits a sharp increase in resistance when the RF signal is turned ON, maintaining a stable resistance during the ON state. Subsequently, a sharp drop in resistance was observed upon turning the RF radiation OFF, returning to its initial resistance value, showing full recovery. This resistance increase can be correlated with temperature rise, where the RF radiation stimulates the free electrons in the metallic NNO film to oscillate, generating heat within the material and thereby increasing the resistance [27]. To further understand this mechanism, we analyzed the average resistance change with RF radiation as a function of temperature (Figure 3f). We found that the average change in resistance [(${\Delta R)}_{avg}$] remained constant at approximately 0.38±0.03 Ω across all measured temperatures. As previously shown (Figure 2b), NNO exhibits metallic behavior in the measured temperature range, with a slope (ΔR/ΔT) of 0.13 Ω/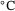. Given this, the ΔR of 0.38 Ω induced by RF radiation corresponds to an estimated temperature increase of $∼3^{\circ }C$ at each measured temperature. Therefore, the observed RF‐induced resistance change can be likely attributed to a heating mechanism. Similar reversible resistance modulations due to temperature variations under different light wavelengths have also been reported for NNO films in the literature [10].

Figures 3g–3j depict the RPCR of the NNO film at $25^{\circ }C$ with varying RF ON/OFF switching ratios, set at 0.1, 0.5, 1, and 10, respectively. The upper panels in Figure 3g–j illustrate the RF ON/OFF durations for different switching ratios, repeated over 10 cycles. These switching ratios were modulated across two orders of magnitude by adjusting the ON/OFF times as follows: 0.1 (0.5 min ON/5 min OFF), 0.5 (1 min ON/2 min OFF), 1 (1 min ON/1 min OFF), and 10 (5 min ON/0.5 min OFF). In all cases, the film demonstrated a highly reversible resistance change, showing its potential for industrial applications such as RF sensors, wireless switches, volatile memories, and reconfigurable electronics.

**FIGURE 4:**
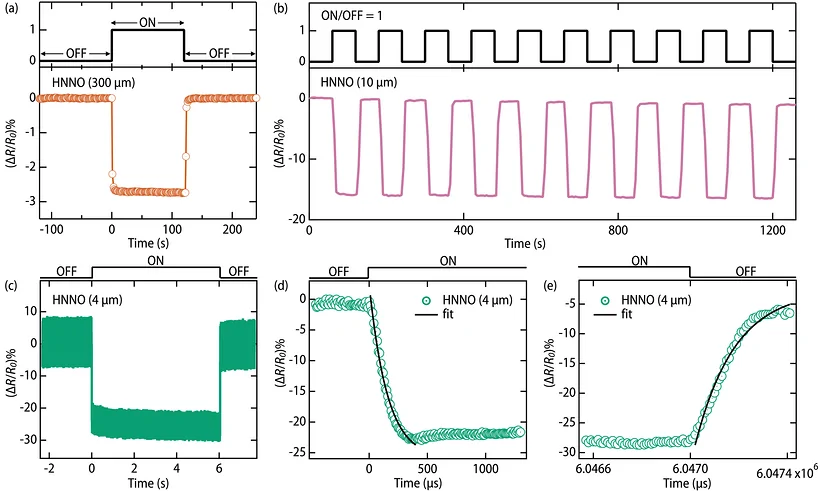
Interaction of RF radiation with HNNO film at room temperature and high‐speed measurement of this interaction. (a) RPCR of the HNNO film with an electrode separation of 300 μm under 2.4 GHz RF radiation at room temperature. A sharp decrease (increase) in resistance is observed when the RF signal is turned ON (OFF). A schematic of the RF ON/OFF states is shown in the upper panel of (a). (b) RPCR of the HNNO film with an electrode separation of 10 μm at room temperature, with ON/OFF ratios of RF radiation set to 1, showing a reversible resistance change under RF radiation. (c) RPCR as a function of time on the microsecond timescale for the HNNO film with an electrode separation of 4 μm under RF radiation. (d,e) Zoomed‐in views of the panel (c) around the RF ON and OFF time, respectively. The black curves represent exponential fits to the data used to extract the time constant.

We further explored the effect of RF radiation on a semiconducting SNO film. Figure S2a,b presents the structural and temperature‐dependent electrical characteristics of the SNO film, confirming its semiconducting behavior at room temperature. Under RF radiation, the RPCR of SNO film decreases upon exposure and fully recovers once the RF signal is turned OFF (Figure S2c), exhibiting reversible resistance modulation similar to that observed in NNO films. The sign of the response is consistent with the semiconducting nature of SNO, where an increase in temperature leads to a decrease in resistance, thereby supporting a thermally driven mechanism. Finally, we extended our investigation to the insulating HNNO film, which exhibits behavior similar to that of SNO (Figure 4a).

The maximum RPCR observed for the HNNO film at room temperature (for electrode gap separation of 300 μm) is approximately −2.7% (negative sign denotes the decrease in RPCR), whereas for the NNO film with the same electrode separation, the maximum RPCR is around +0.3% (Figure 3b). Given that HNNO is insulating (Figure 2c), its resistance is expected to decrease with RF radiation exposure based on the heating mechanism. The reversible resistance change in HNNO under RF exposure for 10 cycles with an ON/OFF switching time ratio of 1 at room temperature is shown in Figure 4b. For this measurement, the electrode separation was 10 μm, resulting in a maximum RPCR of −16%. A reversible resistance change for 10 cycles of an HNNO film with a 300 μm electrode separation is shown in Figure S3.

Additionally, we performed time‐resolved measurements recording resistance values at 10‐μs intervals for the HNNO film with an electrode separation of 4 μm (Figure 4c), which exhibits an enhanced maximum RPCR of approximately −30%. From measurements across different electrode separations, we find that the RPCR magnitude increases with decreasing channel gap. Figure 4d and Figure 4e show the zoom‐in view of Figure 4c around the RF ON and OFF times, respectively. To determine the characteristic time constant (τ) associated with the resistance rise and recovery upon RF exposure, the data in Figure 4d,e were fitted with a single exponential function (black curves). The extracted τ values are approximately 156±8 and 163±12 μs for the resistance drop and rise, respectively. This short time scale sensitivity can likely be further tuned by varying channel separation. This volatile resistance change occurring on the microsecond timescale can be associated with the STP observed in excitatory synapses, where STP represents a transient modification in synaptic strength in response to external stimuli, typically spanning timescales from milliseconds to minutes, and is inherently reversible [28, 29, 30, 31]. Thus, RF radiation acts as a stimulus that induces STP‐like behavior in nickelate thin films.

### 2.3 Visible and Infrared Radiation Driven Intermediate‐Timescale Dynamics

To further explore the effect of electromagnetic radiation across different photon energies, we examined the response of nickelate films under visible (red, 650 nm) and infrared (IR, 3.3 μm) illumination. The resistance responses of NNO, HNNO, and SNO films under visible and IR excitation are shown in Figures S4 and S5, respectively. Under both visible and IR illumination, all films exhibit reversible resistance modulation, where the resistance increases in NNO and decreases in HNNO and SNO during illumination and returns to its initial value once the stimulus is removed. This behavior is similar to that observed under RF excitation and indicates the absence of any persistent or non‐volatile effects. The sign of the resistance change is consistent with the intrinsic transport characteristics of the films, supporting a thermally driven mechanism. Similar resistance changes arising from thermally driven effects under visible‐light illumination have been previously reported in NNO [10, 11]. All visible and IR measurements were performed with the chiller turned on, and the corresponding details are discussed in the following section.

The relaxation data after removal of the stimulus were fitted using a single exponential function to extract the τ. The extracted τ values are on the order of seconds under visible illumination (1.74±0.17 s for NNO, 1.9±0.92 s for HNNO, and 1.38±0.49 s for SNO) and increase to tens of seconds under IR illumination (59.58±0.73 s for NNO, 48.17±0.74 s for HNNO, and 70.66±1.2 s for SNO). These timescales fall in the behavioral regime and are longer than those observed under RF excitation, indicating a slower but still reversible relaxation process.

### 2.4 UV‐Induced Non‐Thermal Effects and Glass‐Like Relaxation

**FIGURE 5:**
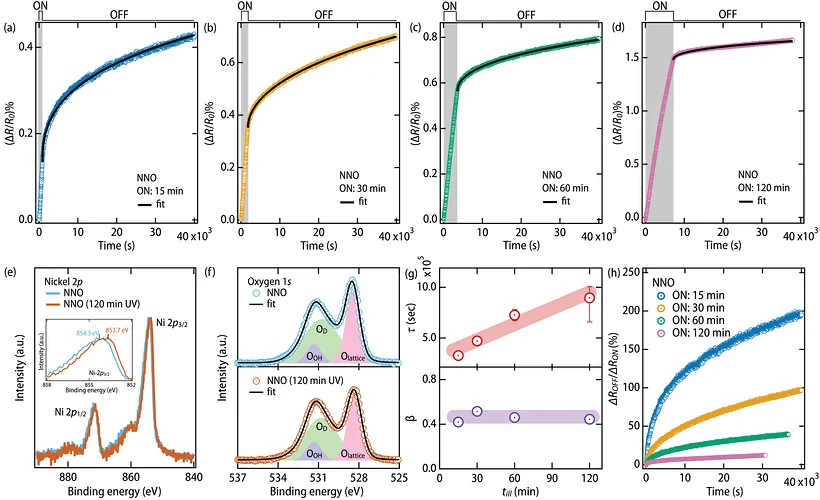
Interaction of UV light with NNO film and aging behavior at $35^{\circ }C$. (a–d) RPCR of the NNO film under UV illumination (254 nm) for durations of 15, 30, 60, and 120 min, respectively. In each case, resistance relaxation is observed after the UV light is turned OFF. The black curves represent stretched exponential fits in the light‐OFF state. (e) Ni‐2p XPS spectra of the NNO film after 120 min of exposure to 254 nm UV light compared to the pristine NNO film. A magnified view of the Ni‐2p 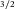 region is presented in the inset. (f) O‐1s XPS spectra of the pristine NNO film (top panel) and the NNO film after 120 min of exposure to 254 nm UV light (bottom panel), fitted with three components corresponding to lattice oxygen ($O_{\mathrm{lattice}}$, pink), oxygen defects ($O_{D}$, green), and hydroxyl groups ($O_{\mathrm{OH}}$, purple). (g) Stretched exponent (β) and relaxation time (τ) extracted from the fitting of panels (a–d) as a function of illumination time ($t_{ill}$). The associated error bars are obtained from the fitting. (h) Change in resistance relaxation in the OFF state for different illumination durations as a function of time normalized by the total resistance drop ($\Delta R_{ON}$) during the ON‐state to show the aging phenomena.

We then studied the influence of short‐wavelength UV light (254 and 365 nm) on electrical properties and temporal dependence in nickelate films. Figure 5a–d depicts the temporal evolution of RPCR in NNO films exposed to 254 nm UV light for 15, 30, 60, and 120 min, followed by the subsequent relaxation phase when the UV light is turned OFF. For all cases, the sample was held at $35^{\circ }C$ for an extended period of time to ensure thermal stability before being exposed to UV light for different time intervals. Initially, the resistance increases upon UV exposure, indicating a negative photoconductivity response. This phenomenon is relatively uncommon and has been attributed to various mechanisms in the literature, including trap states, surface adsorption effects, and plasmonic interactions [32]. Additionally, a non‐volatile resistance change is observed after the UV light is switched off, where the resistance remains elevated rather than returning to its original value. A recent study has reported giant persistent photoconductivity extending over 100 h at room temperature in Sn‐based perovskites [33]. This persistent resistance change suggests a mechanism beyond simple thermal effects, since a purely thermal response would be reversible eventually as the sample reverts to its initial state, as noted in the previous section of RF exposure.

To further confirm the non‐thermal nature of the UV response in nickelate films, we conducted two additional experiments: (i) measuring RPCR in an NNO film with the chiller system switched ON and OFF (see Figure S6), and (ii) investigating the effect of UV exposure on a SNO film (see Figure S7). Figure S6a illustrates the resistance and temperature response of an NNO film at room temperature under cyclic exposure to 254 nm UV light when the chiller was turned OFF. In this scenario, resistance increased upon UV illumination and decreased when the UV light was turned OFF. Simultaneously, the temperature fluctuations exhibited a similar trend, rising during UV exposure and dropping once the UV light was turned OFF. Since NNO is metallic at room temperature, this behavior can be attributed to a thermal effect, where UV radiation induces gentle heating in the material, leading to an increase in resistance, followed by a cooling‐driven resistance decrease upon turning OFF the UV light. In contrast, Figure S6b shows the resistance response of the NNO film under UV exposure while the chiller was turned ON. This experiment was conducted at a stabilized temperature of $35^{\circ }C$, with the chiller actively maintaining a constant thermal environment. Notably, while the film's resistance still increased upon UV illumination, it did not decrease after the UV light was switched OFF. The absence of a resistance drop despite no measurable temperature variation indicates that the observed effect is not purely thermal in origin. Furthermore, Figure S7 presents the response of an SNO film to 254 nm UV light exposure. The SNO film exhibits semiconducting behavior at $35^{\circ }C$, as verified by temperature‐dependent resistance measurements (see Figure S2b). If the UV‐induced effect were solely thermal, an increase in temperature should lead to a decrease in resistance. However, the experimental data reveal the opposite trend—an increase in resistance upon UV exposure (see Figure S7). This further validates the hypothesis that the effect of UV light on the resistance of SNO is not due to heating alone but instead arises from a non‐thermal mechanism. The observed persistent increase in resistance suggests that UV exposure induces material modifications that require further investigation and are discussed below.

To verify whether UV exposure produced any nanoscale electronic inhomogeneity, we performed scattering‐type scanning near‐field optical microscopy (s‐SNOM) on pristine and UV‐treated NNO films. The nano‐infrared near‐field amplitude maps obtained at an incident light frequency of 1000 ${\mathrm{cm}}^{-1}$ show that both samples remain homogeneous at nanoscale, and the UV‐treated film exhibits no detectable contrast relative to the pristine film, consistent with its minimal resistance change (see Figure S8). The spatial uniformity of the near‐field response indicates that the UV‐induced resistance increase does not originate from nanoscale phase separation or disorder, but instead may arise from a uniform modification of the underlying stoichiometry. A possible mechanism may be due to the formation of oxygen vacancies under UV illumination, a phenomenon that has been reported in various correlated oxides [34, 35, 36, 37, 38]. The formation of oxygen vacancies is known to increase the resistance in nickelates [39]. The metallic nature of NNO above MIT originates from the hybridization of Ni‐3d and O‐2p orbitals, where nickel predominantly has a 3+ valence state. The formation of oxygen vacancies introduces extra electrons into the system, reducing some of the Ni from a 3+ to a 2+ oxidation state [40]. The larger radius of the ${\mathrm{Ni}}^{2+}$ ion increases the Ni‐O bond length and reduces the Ni‐O‐Ni bond angle as the tolerance factor increases, and resulting in a more resistive phase.

Building on the observation for UV‐induced non‐volatile resistance changes in NNO film, we examine the microscopic origin of these effects by probing the changes in the chemical environment via x‐ray photoelectron spectroscopy (XPS) measurements. We have assessed the oxidation state of Ni and the oxygen content in the films following UV exposure. Figure 5e shows the Ni‐2p core‐level XPS spectra of both pristine NNO film and NNO after 120 min of 254 nm UV illumination. In the pristine state, the Ni‐2p 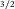 peak is centered at approximately 854.3 eV, which gradually shifts to 853.7 eV after UV exposure of 120 min (inset of Figure 5e). A similar shift of 0.6 eV is observed in the Ni‐2p 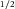 peak, moving from 871.9 to 871.3 eV. This systematic shift to lower binding energies indicates a reduction in the oxidation state of Ni, consistent with a partial transition from ${\mathrm{Ni}}^{3+}$ to ${\mathrm{Ni}}^{2+}$ [23, 41]. To further substantiate the presence of oxygen vacancies, we analyze the evolution of the O‐1s core‐level spectra shown in Figure 5f. The spectra were fitted with three components corresponding to lattice oxygen ($O_{\mathrm{lattice}}$) at ∼528.5 eV, oxygen defects ($O_{D}$) at ∼530.8 eV, and hydroxyl groups ($O_{\mathrm{OH}}$) at ∼531.35 eV [23]. The area corresponding to $O_{\mathrm{OH}}$ remained nearly unchanged. To qualitatively evaluate the generation of oxygen vacancies, we calculated the ratio of the integrated area of the oxygen defect peak to that of the lattice oxygen peak [area($O_{D}$)/area($O_{\mathrm{lattice}}$)]. This ratio increased from 1.5 in the pristine NNO film to 1.61 after UV exposure, indicating a small increase in oxygen defect concentration. These XPS results suggest that UV radiation induces a reduction of nickel ions due to the formation of oxygen vacancies in NNO films, which collectively contribute to the observed increase in electrical resistance upon UV exposure. In contrast, RF exposure does not produce any measurable shift in the Ni‐2p peak, indicating that the Ni valence state remains unchanged (see Figure S9). This suggests that the reversible resistance change observed under RF exposure does not arise from oxygen vacancy formation. We also measured representative current–voltage (I–V) characteristics of NNO, HNNO, and SNO devices at room temperature before and after UV exposure, as shown in Figure S10. All devices exhibit linear I–V behavior, confirming ohmic contacts.

Further, we examined the effect of photon energy by exposing an NNO film to a 365 nm UV source for 30 min (see Figure S11). The RPCR increased with UV illumination, similar to the response observed under 254 nm exposure; however, the magnitude of the change was lower for 365 nm than for 254 nm. We found that a 30‐min exposure to 254 nm UV light resulted in an RPCR of 0.35% (Figure 5b), while exposure to 365 nm UV light yielded a significantly lower RPCR of 0.07% (Figure S6). This observation demonstrates that resistance change diminishes with increasing UV light wavelength, a behavior consistent with literature observations [10, 12].

We fitted the RPCR relaxation curves corresponding to different UV illumination times ($t_{ill}$) using a stretched exponential function of the form: $a+b\times e^{-{\left(t/\tau \right)}^{\beta }}$, where a and b are fitting constants, τ represents the characteristic relaxation time, and β is the stretched exponent. The fitted curves with stretched exponential are shown as black lines in Figure 5a–d. Notably, the stretching exponent β was found to be a constant around 0.45±0.05 (lower panel of Figure 5g), where 0 < β < 1. Such stretched exponential relaxation behavior typically indicates the presence of multiple relaxation pathways and is often considered a signature of glassy dynamics [42]. Similar relaxation characteristics have been observed in a variety of disordered and glassy systems in the literature [43, 44, 45, 46, 47]. While glass‐like behavior and slow relaxations have been reported in neodymium nickelate near the MIT critical point (around 200K) [7, 8, 9, 48, 49], our study presents the first experimental demonstration of such behavior in the nominally metallic phase of NNO at room temperature. Moreover, the characteristic relaxation time (τ) was found on the order of 10
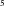
–10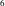 s, which is much larger than the Drude relaxation time ($\tau \approx 10^{-14}-10^{-15}s$ for metals), signifying glass‐like slow relaxation. These observations indicate the emergence of extrinsic electron glass‐like dynamics in our system, as the resistance modification is driven by UV light exposure [43, 46, 50, 51].

Moreover, τ increases systematically with increasing $t_{ill}$ (upper panel of Figure 5g), which is a characteristic signature of aging phenomena. Aging is a key criterion for glassy behavior and refers to the system's dependence on its history, where older systems exhibit slower relaxation dynamics compared to younger ones [45, 52, 53]. To further illustrate this effect, Figure 5h presents the resistance relaxation in the OFF‐state as a function of time for different $t_{ill}$. As the amount of light received by the film depends on $t_{ill}$, the change in resistance in the OFF state ($\Delta R_{OFF}=R-R_{f}$, where $R_{f}$ is the resistance when the light is turned OFF) is normalized with a total change in resistance during the ON state ($\Delta R_{ON}$). It is evident that with increasing illumination time, the relaxation dynamics become progressively slower (upper panel of Figure 5h), indicating a more sluggish system response. Additionally, the extracted τ values from the fitting analysis exhibits a linear scaling with $t_{ill}$, (upper panel of Figure 5g), indicating aging behavior [45, 52, 53]. In summary, our findings provide evidence of glass‐like relaxation coupled with aging behavior in the metallic phase of NNO films triggered by UV illumination.

### 2.5 Non‐Associative Learning Along With Habituation and Sensitization Behaviors Under Repetitive UV Stimuli

**FIGURE 6:**
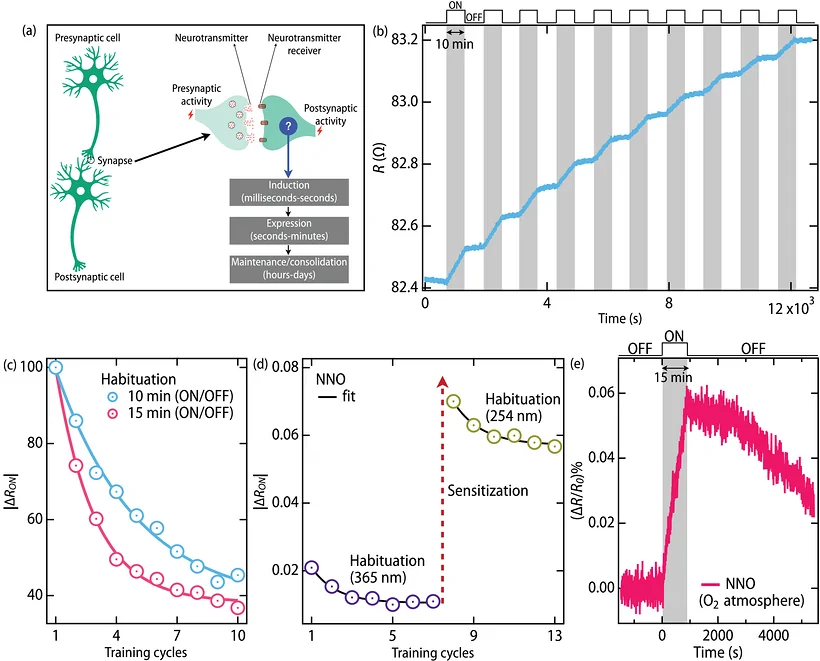
Schematic of neurons, demonstration of habituation and sensitization behavior of NNO with UV light at $35^{\circ }C$. (a) An illustration of a synapse between two neurons, highlighting the three phases of synaptic plasticity with different time scales. Pre‐ and postsynaptic activity initiates plasticity induction, leading to physical expression of the changes in synaptic strength. These changes are later stabilized or further modified during maintenance and consolidation. (b) Resistance of the NNO film during alternating exposure to UV light (254 nm) for 10 min ON and 10 min OFF, repeated over 10 training cycles. (c) Normalized absolute values of the resistance change as a function of training cycles for different pulse durations (10 and 15 min ON/OFF) calculated from panel (b) and Figure S13, respectively. The solid curves represent exponential fits to the data, demonstrating pulse duration‐dependent habituation behavior. (d) RPCR of the NNO film under repeated 365 nm UV illumination with 5 min ON/OFF durations for 7 training cycles, showing clear habituation behavior. Upon switching to 254 nm UV exposure in the 8th cycle, the response amplitude increases beyond that of the initial 365 nm cycle, demonstrating sensitization. Subsequent repeated 254 nm UV exposure with 5 min ON/OFF durations for 6 cycles again exhibits habituation behavior. (e) RPCR of the NNO film under 254 nm UV illumination for 15 min in an oxygen‐rich environment.

Similar to pristine NNO, where τ lies in the order of 10
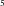
–10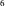 s (Figure 5g), the stretched exponential fit applied to the relaxation data of an HNNO film exposed to UV light for 60 min yielded a τ of (1.44$\pm 0.1)\times 10^{4}$ and a β of 0.51±0.004 (See Figure S12). These long relaxation timescales in nickelate films can be analogous to the slow learning processes observed in biological systems. Figure 6a illustrates a schematic of a synapse, at the junction between two neurons. When a signal reaches the presynaptic terminal, it triggers the release of neurotransmitters across the synaptic gap. These neurotransmitters bind to receptors on the postsynaptic membrane, altering its excitability and triggering an impulse. While Hebbian‐like correlated activity occurs on a millisecond‐to‐second timescale, synaptic plasticity remains sensitive over much longer periods. Synaptic plasticity is typically divided into three phases: induction, expression, and maintenance/consolidation (Figure 6a). Induction is the initial process that links neural activity patterns to a biochemical decision, determining whether a synapse should strengthen, weaken, or remain unchanged. Expression refers to the physical manifestation of synaptic strength changes, which may occur with a delay of seconds to minutes after induction. Maintenance and consolidation involve the long‐term stabilization or modification of these changes, spanning hours to days and potentially persisting for years [1]. For instance, Wiegert et al. [54] demonstrated that potentiated synapses in vitro hippocampal slice cultures can be resistant to depression‐inducing protocols delivered even 24 h later.

The temporal and history‐dependent resistance changes make it possible to demonstrate habituation and sensitization, which are most common forms of non‐associative learning. Habituation is widely studied due to its universal occurrence and its role as an elementary model of adaptive learning [55, 56]. A common property of habituation can be described as repeated stimulation that leads to a decrease of the response [57, 58]. Additionally, habituation often follows an exponential decay [55, 56, 57, 58, 59, 60, 61, 62], which can be described by the function: $a\times e^{-(b\times x)}$, where a is a constant and b expresses the rate of habituation, with a greater value of b corresponds to faster learning. To demonstrate habituation in NNO films, we exposed the sample to ten successive cycles of UV light exposure, alternating between UV ON and OFF states for 10 min each. Figure 6b shows the resistance (R) change during these cycles, while Figure 6c illustrates the absolute values of the resistance change during ON‐state (defined as $\Delta R_{ON}$=$R-R_{i}$, where $R_{i}$ is initial resistance of that specific cycle) as a function of training cycles. The observed decrease in resistance change over repeated stimulation Figure 6c demonstrates habituation. To establish pulse‐dependent plasticity, we then investigated the effect of pulse duration by performing habituation measurements using 10 successive cycles of UV illumination with 15 min ON/OFF intervals, as shown in Figure S13. Figure 6c compares the normalized habituation behavior for different pulse durations (10 and 15 min ON/OFF cycles). The data were fitted using an exponential function, yielding fitting parameters b = 0.25±0.03 and 0.52±0.03 for the 10 and 15 min ON/OFF cycles, respectively. The increase in b with longer pulse duration indicates faster decay of the response, demonstrating clear pulse‐duration‐dependent habituation behavior. In addition to habituation, we observe sensitization behavior under repeated UV exposure, where sensitization is defined as an enhancement of the response amplitude upon application of a new or stronger stimulus. Figure 6d shows habituation under repeated 365 nm UV illumination with 5 mins ON/OFF cycles. Upon switching to a higher‐energy 254 nm UV stimulus, the response amplitude increases, demonstrating sensitization, followed by a subsequent decrease with repeated 5 mins ON/OFF cycles, indicating habituation under the new stimulus.

Finally, to directly probe the role of oxygen vacancies, we performed UV exposure (15 min) experiments under different environments, including Ar, ambient air, and oxygen‐rich conditions. The resistance change is largest in Ar (RPCR ≈ 0.29%, see Figure S14), indicating that the observed modulation cannot be attributed solely to surface adsorption or oxidation. In ambient air, the resistance change is reduced (RPCR ≈ 0.18%, see Figure S15) but remains comparable to previously observed values (Figure 5a), confirming the reproducibility of the effect. In contrast, the response is significantly suppressed in an oxygen‐rich environment (RPCR ≈ 0.05%), with partial recovery upon removal of the UV stimulus (Figure 6e), consistent with oxygen re‐incorporation. These results provide strong evidence for a defect‐mediated mechanism, where UV‐induced oxygen vacancy formation governs the resistance modulation. Additionally, the recovery behavior in oxygen represents a form of spontaneous recovery, further demonstrating another characteristic of learning behavior in the system.

**FIGURE 7:**
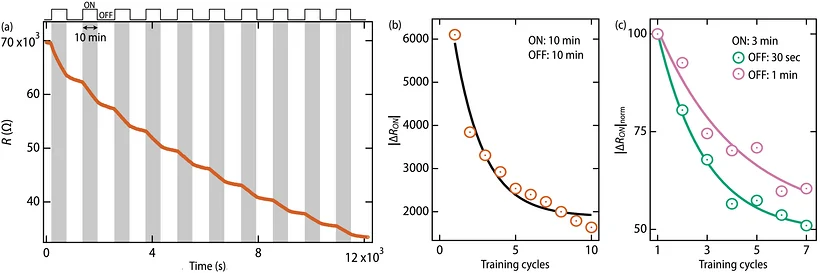
Habituation behavior of HNNO with UV light at $35^{\circ }C$. (a) Resistance of the HNNO film under repeated exposure to UV light (254 nm) with 10 min ON and 10 min OFF cycles, conducted over 10 training cycles. (b) Absolute values of the resistance change in each cycle, extracted from panel (a), as a function of the training cycle. The black curves represent an exponential fit to the data, demonstrating habituation behavior. (c) Normalized absolute values of the resistance change of the HNNO film as a function of the training cycle during alternating UV exposure for 3 min ON, followed by 30 s and 1 min OFF periods, repeated over 7 training cycles. The solid curves represent an exponential fit to the data. With a 30 s OFF state, the UV light stimulus is more frequent, resulting in a faster decline in resistance change over the training cycles.

Figure 7a depicts the resistance response of HNNO under similar successive UV light exposure condition. The absolute resistance change as a function of training cycles was fitted with an exponential function with b = 0.52±0.08 (Figure 7b), demonstrating habituation in HNNO film as well. Additionally, another key property of habituation states that, “The more frequent the stimulus presentation, the more rapid and pronounced the decrease” [56, 57, 58]. To test this, we varied the UV exposure frequency while maintaining a fixed ON time of 3 min but altering the OFF time between cycles. In case 1, the OFF time was set to 30 s, while in case 2, it was 1 min, resulting in a higher stimulus frequency in case 1. The exponential fits to these datasets yielded b = 0.52±0.07 and 0.31±0.1 for the two cases, respectively (Figure 7c). Both datasets are normalized to enable better comparison. The faster decay in case 1 with higher b value shows that increased stimulus frequency accelerates the habituation response, provides additional validation for learning behavior in the nickelate films.

### 2.6 Memory‐Based Dynamical Modeling of Stimulus‐Driven Learning

Motivated by recent work of modeling habituation in biological systems [63] and analog circuits [64], we developed minimal dynamical models that capture key features of UV‐induced resistance changes in NNO (see Supporting Information Text, Figure S16a). We consider a family of ordinary differential equations involving linear memory variables that record the history of UV exposure and nonlinearly modulate the resistance dynamics during the ON and OFF phases. Using just two memory variables with different timescales: a fast one representing more recent stimulation and a slow one encoding cumulative exposure history—the model can account for the slow relaxation with increased exposure reported in Figure 5a–d,h (see Figure S16b,c) as well as the habituation of resistance gain in response to pulsatile inputs shown in Figure 6 (see Figure S16d,e). These results provide an initial mathematical framework for understanding stimulus‐dependent learning in nickelates and illustrate the utility of such memory‐based approaches for modeling complex material responses to dynamic stimuli.

## 3 Conclusion

We have examined the electrical response of several perovskite nickelate systems spanning metals to insulators under electromagnetic radiation across a broad frequency range, including RF, IR, visible, and UV radiation. Our findings establish that RF radiation induces short‐term plasticity, whereas visible and infrared illumination give rise to reversible resistance changes on behavioral timescales. In contrast, UV exposure induces persistent long timescale memory effects in the same material system. We observed glass‐like relaxation dynamics under UV illumination, characterized by stretched exponential behavior and aging phenomena, along with additional learning behaviors such as habituation, sensitization, and spontaneous recovery under controlled environments. The ability to tune the response through pulse duration, frequency, and stimulus strength further demonstrates pulse‐dependent plasticity in these systems. A minimal dynamical systems model captures key features of the complex temporal dynamics, providing a qualitative framework for understanding stimulus‐dependent learning in nickelates. These insights open new avenues to study glass physics in nickelates without being restricted to thermal phase transition boundary limits and suggest avenues to design multi‐timescale responsive opto‐electronic components for emerging artificial intelligence hardware.

## 4 Experimental Section

### 4.1 Film Growth

NNO films of thickness ∼50 nm were grown on (100)‐oriented LAO substrates (10 mm ×10 mm in dimension) using a radio‐frequency (RF) magnetron sputtering system via co‐sputtering of high‐purity (99.9%) Nd and Ni targets. During deposition, an RF power of 135 W and a DC power of 75 W were applied to the Nd and Ni targets, respectively. Prior to film growth, the targets were pre‐sputtered for 10 min. The deposition was carried out at room temperature under a controlled pressure of 5 mTorr, maintained by introducing 30 SCCM (standard cubic centimeters per minute) of Ar and 10 SCCM of $O_{2}$ gases. To ensure uniform film thickness and composition, the substrate holder was continuously rotated at 40 rpm during deposition. Post‐deposition, the films were annealed in a furnace at $500^{\circ }C$ for 24 h in air. Subsequently, a 50 nm Pd layer was deposited using e‐beam evaporation through a shadow mask, serving both as electrical contacts and as a catalyst for hydrogen incorporation [16, 21, 23]. Hydrogen‐doped NNO (HNNO) films were synthesized by annealing the NNO film with Pd electrodes in a furnace at $150^{\circ }C$ for 30 min in a forming gas environment (5% $H_{2}$/95% Ar). For the deposition of SNO film, an Sm target was utilized with an RF power of 150 W, while maintaining all other growth parameters identical to those used for NNO film deposition.

### 4.2 Structural and Electrical Characterization

Structural characterization of the films and the substrate were performed using x‐ray diffraction (XRD) with a Panalytical X'Pert diffractometer equipped with a Cu source. The dc electrical transport measurements were carried out in a two‐probe configuration using an INSTEC HP1000V‐PM probe station with Keithley 2635A and 2400 source meters. The temperature was controlled by an INSTEC mK2000 precision temperature controller (see Figure S1 for the experimental setup). Electrical contacts were made by directly probing the metal electrodes with micromanipulator tips, and resistance was measured by applying a bias voltage of +0.05 V.

### 4.3 RF, IR, Visible, and UV Measurements

For RF measurements, an X86‐based Software Defined Radio (SDR) platform was utilized in combination with a B210 Universal Software Radio Peripheral (USRP) and a directional antenna [65]. A 2.4 GHz RF signal with a power of 11.1 dBm was used for the RF exposure experiments, employing a narrowband sine wave as the waveform. The directional antenna, coupled with a power amplifier, focused the RF energy onto the samples from a distance of 8 cm. Time‐resolved high‐speed measurements on HNNO films were conducted using a Keithley 2461 source meter by applying a constant voltage of +0.05 V. IR illumination was produced by using an LED source with a wavelength of 3.3 μm, operated at a forward bias voltage of 2.2 V, delivering an output power of 0.8 mW. Visible light was generated by using a red laser (650 nm) with an output power of 5 mW. Handheld UV lamps with 254 and 365 nm were used for UV illumination studies. The lighting intensities were 22.5 μW/${\mathrm{mm}}^{2}$ for the 254 nm lamp and 10 μW/${\mathrm{mm}}^{2}$ for the 365 nm lamp. The power of both lamps were 6 W. The distance between the samples and the source was 2 cm for IR, visible, and UV radiation experiments. See Figure S1 for the experimental setup. For RF measurements on NNO, the resistance modulation is fully reversible, and all measurements were performed on the same device. For HNNO, RF measurements were carried out on devices with different electrode separations (300, 10, and 4 μm) to examine geometry‐dependent effects. In contrast, UV‐induced resistance changes are non‐volatile. Hence, each measurement was performed on a separate film grown under identical conditions in the same run to ensure reproducibility.

### 4.4 SNOM

Infrared nano‐imaging was carried out using scattering‐type scanning near‐field optical microscopy (s‐SNOM) illuminated by continuous‐wave mid‐infrared quantum cascade lasers. An atomic force microscope (AFM) tip was used in this technique with an apex radius of ∼20 nm, operating in tapping mode at ∼270 kHz. Infrared light was focused onto the tip using a parabolic mirror, concentrating the optical field into a nanoscale (∼20 nm) near‐field “hot spot” at the tip apex. When the sample is positioned within this confined optical field, the tip–sample pair forms a coupled optical antenna. Their near‐field interaction modifies the light field scattered back from the tip, and the resulting scattered field encodes critical information about the local complex dielectric function of the sample under study. To isolate the genuine near‐field component from the much stronger background far‐field scattering, the scattered signal was demodulated at the higher harmonic of the tip‐tapping frequency and measured using a pseudo‐heterodyne interferometric detection scheme [66]. This approach not only eliminates far‐field background but also simultaneously provides access to both the near‐field amplitude s(ω) and phase ϕ(ω) of the complex near‐field signal [67, 68]. For quantitative near‐field analysis across samples, a reference measurement of the Pd electrode was included in each near‐field image. Using the nano‐IR signal of Pd ($s_{Pd}$) as a reference, the NNO response was normalized according to: s(ω) = $s_{NNO}$/$s_{Pd}$, following Refs. [67, 69].

### 4.5 XPS

X‐ray photoelectron spectroscopy (XPS) measurements were carried out using a Thermo K‐Alpha instrument with a monochromatized Al‐Kα x‐ray source and a spot size of 400 μm.

## Conflicts of Interest

None of the authors have a conflict of interest to disclose.

## Supporting information


**Supporting File**: advs75984‐sup‐0001‐SuppMat.pdf

## Data Availability

The data that support the findings of this study are available from the corresponding author upon reasonable request.
