# Supplementary material for: Electromagnetic Radiation Stimulated Learning in Perovskite Nickelates
Source: Adv Sci (Weinh). 2026 Jun 12:e75984. Online ahead of print. doi: 10.1002/advs.75984 (PMC13336518; doi:10.1002/advs.75984)
Supplement: Supplementary file 1 — Supporting File: advs75984‐sup‐0001‐SuppMat.pdf [file ADVS-9999-e75984-s001.pdf]

Supplementary Materials

Electromagnetic radiation stimulated learning in perovskite nickelates

Ranjan Kumar Patel,[1,*] Kabir Zama,[1] Matthew Smart,[2] Ramya Eathirajan,[3,4] Ivan Seskar,[5] Narayan Mandayam,[5] Guangxin Ni,[3,4] Martin Mönnigmann,[6] and Shriram Ramanathan[1,†]

[1]*Department of Electrical and Computer Engineering, Rutgers University, Piscataway, NJ 08854, USA*

[2]*Lewis-Sigler Institute of Integrative Genomics, Princeton University, Princeton, NJ 08540, USA*

[3]*Department of Physics, Florida State University, Tallahassee, Florida 32306, USA*

[4]*National High Magnetic Field Laboratory, Tallahassee, Florida 32310, USA*

[5]*WINLAB, Rutgers University, North Brunswick, NJ 08902, USA*

[6]*Department of Mechanical Engineering, Ruhr University Bochum, Bochum 44801, Germany*

[*] ranjan.patel@rutgers.edu

[†] shriram.ramanathan@rutgers.edu

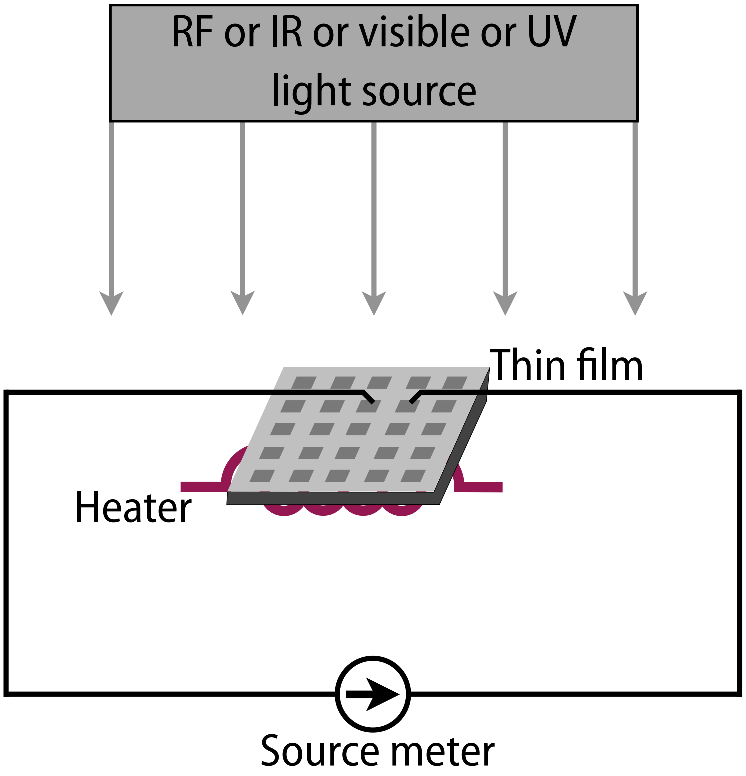

FIG. S1. **Experimental setup.** A schematic illustration of the experimental setup for electrical measurements under varying frequencies exposure. A heater is used for high temperature measurements.

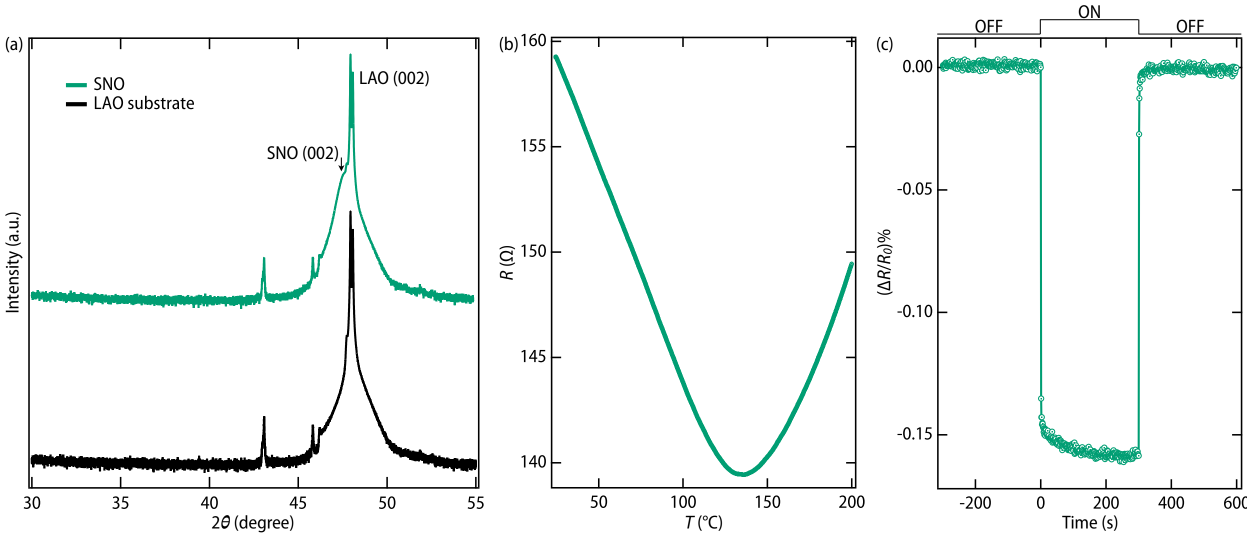

FIG. S2. **Interaction of RF radiation with SNO film at 25°C** (a) XRD data of SNO films deposited on an LAO substrate, accompanied by a reference spectrum of the LAO substrate. Film peaks are indicated with (↓). Data have been vertically offset along the intensity axis for clarity. (b) Temperature-dependent resistance measurements of SNO films, showing semiconducting behavior at room temperature. (c) RPCR of the SNO film under RF radiation of 2.4 GHz at 25°C, demonstrating reversible resistance modulation consistent with a thermally driven mechanism.

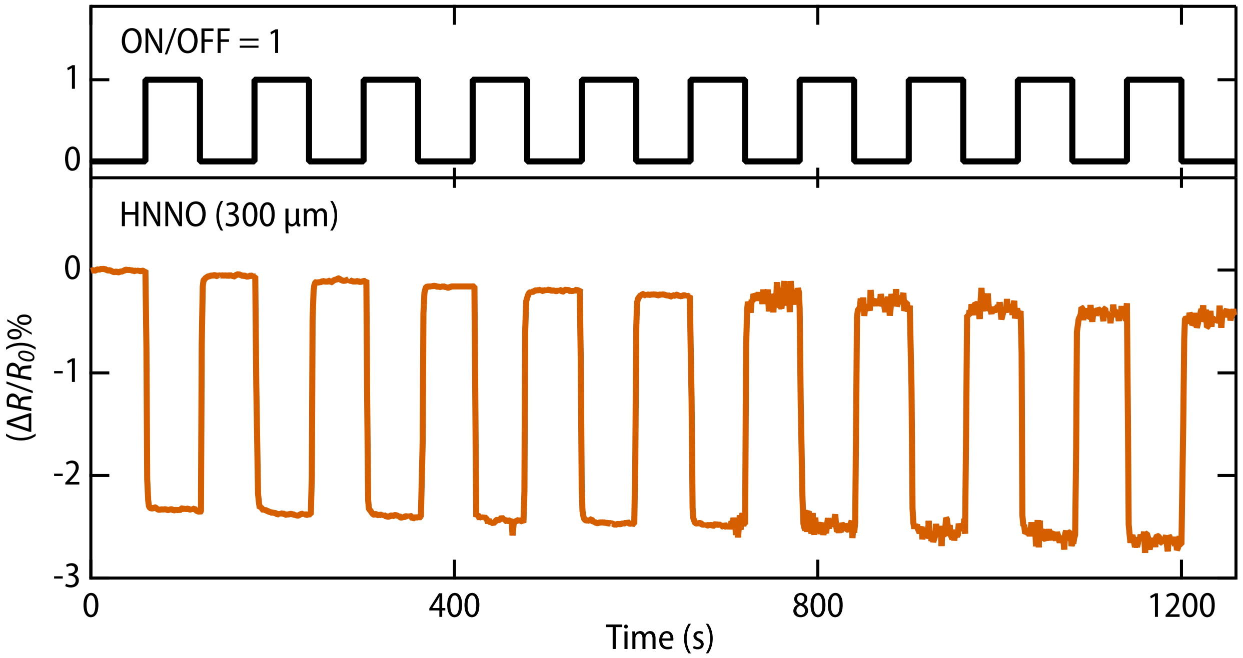

FIG. S3. **Reversible resistance change for HNNO film at 25°C.** RPCR of the HNNO film with an electrode separation of 300 μm at 25°C, showing a reversible resistance change under RF radiation with an ON/OFF ratio of 1. A schematic representation of the RF ON/OFF states is shown in the upper panel.

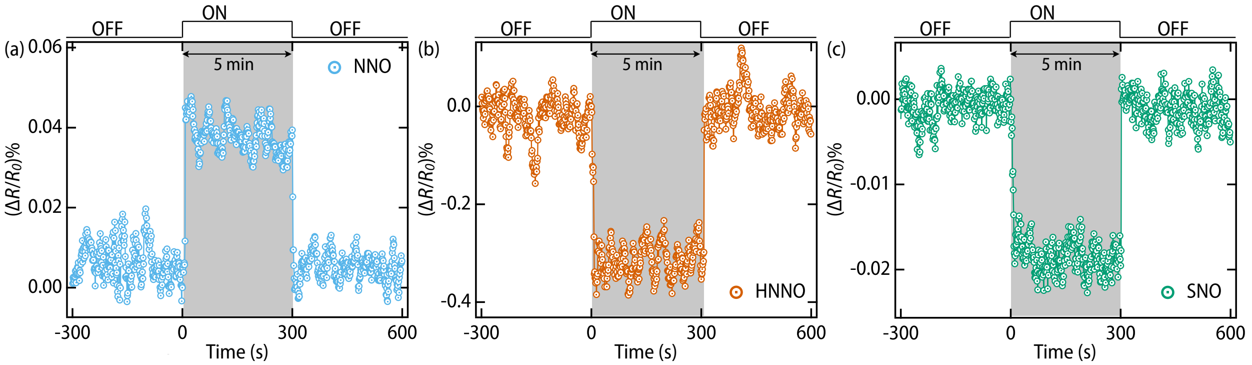

FIG. S4. **Interaction of visible light (red, 650 nm) with nickelate films at 35°C.** (a–c) RPCR of the NNO, HNNO, and SNO films under 650 nm red light illumination for 5 mins at 35°C, respectively. All films exhibit reversible resistance changes with relaxation timescales on the order of seconds, indicating a predominantly thermal response.

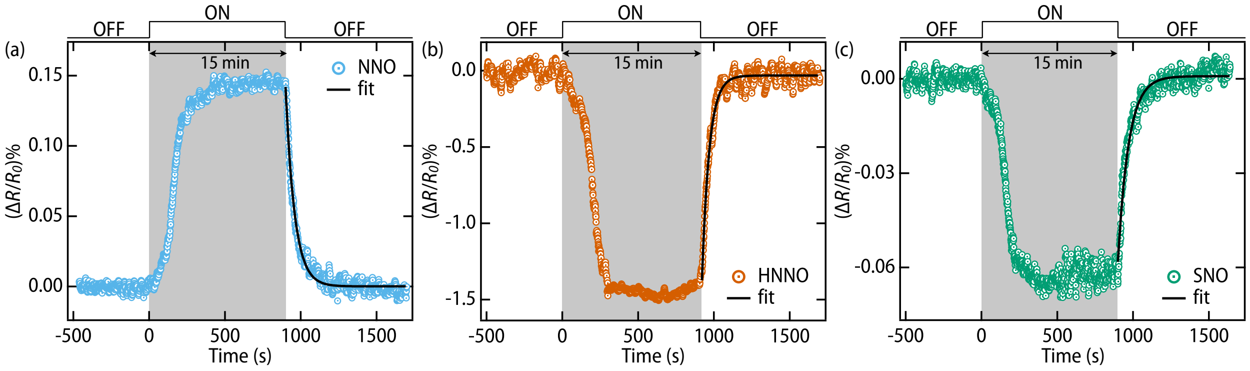

FIG. S5. **Interaction of IR light (3.3 μm) with nickelate films at 35°C.** (a–c) RPCR of the NNO, HNNO, and SNO films under 3.3 μm infrared (IR) illumination for 15 mins at 35°C, respectively. All films exhibit reversible resistance changes with relaxation timescales on the order of seconds, indicating a predominantly thermal response.

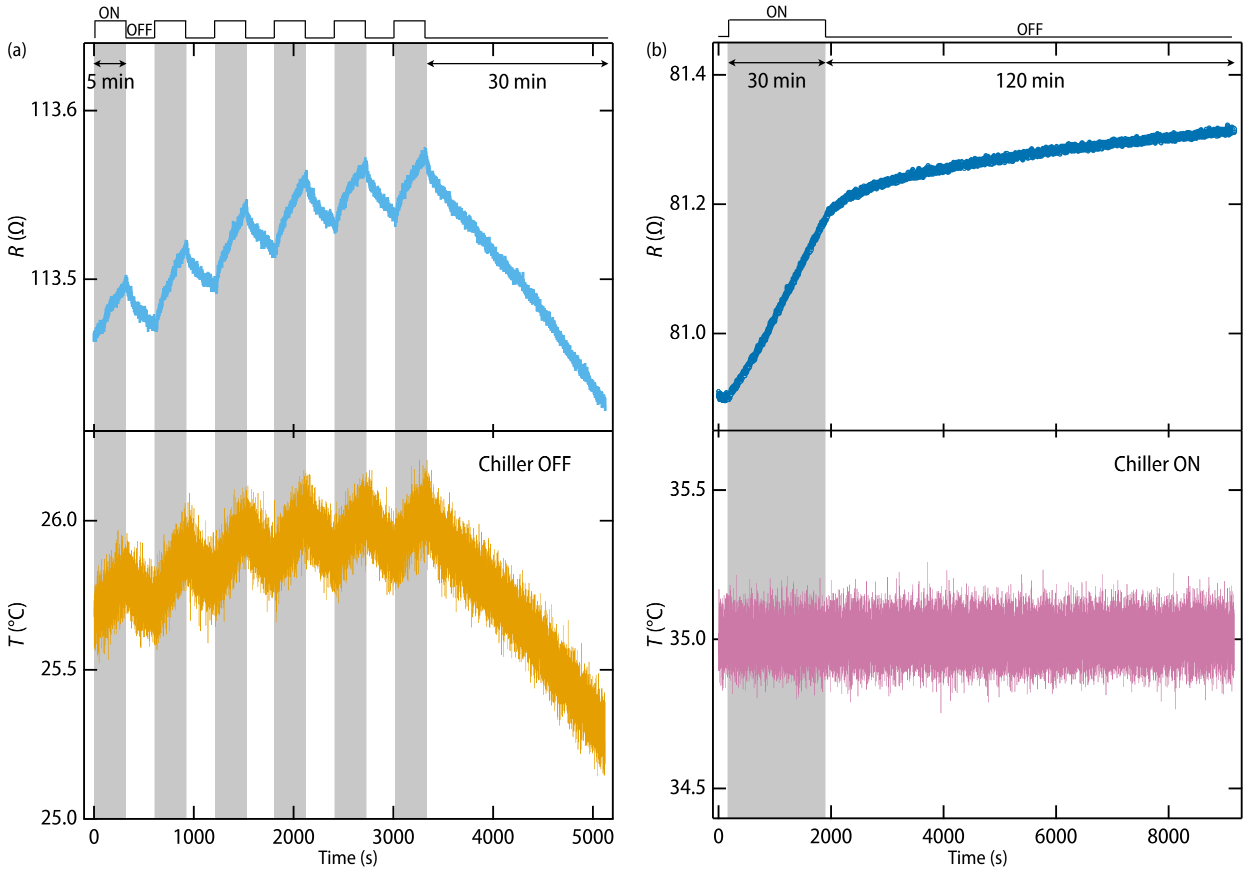

FIG. S6. **254 nm UV exposure on NNO film with and without turning ON the chiller.** (a) Variation in resistance and temperature of the NNO film near room temperature under cyclic UV exposure, with 5 mins ON and 5 mins OFF intervals, repeated over six cycles while the chiller remains OFF. The top panel of (a) illustrates the increase (decrease) in resistance corresponding to the UV light being switched ON (OFF). The bottom panel of (a) displays the simultaneous increase (decrease) in stage temperature when the UV light is turned ON (OFF). Since the NNO film exhibits metallic behavior at room temperature, the observed resistance changes can be attributed to temperature variations. (b) Resistance and temperature response of the NNO film at 35°C under continuous UV exposure for 30 minutes while the chiller remains ON. The bottom panel of (b) shows a stable stage temperature despite UV illumination, as the chiller effectively dissipates the heat generated by the UV light. The top panel of (b) reveals an increase in resistance under UV exposure, indicating that the resistance change is not solely driven by temperature effects.

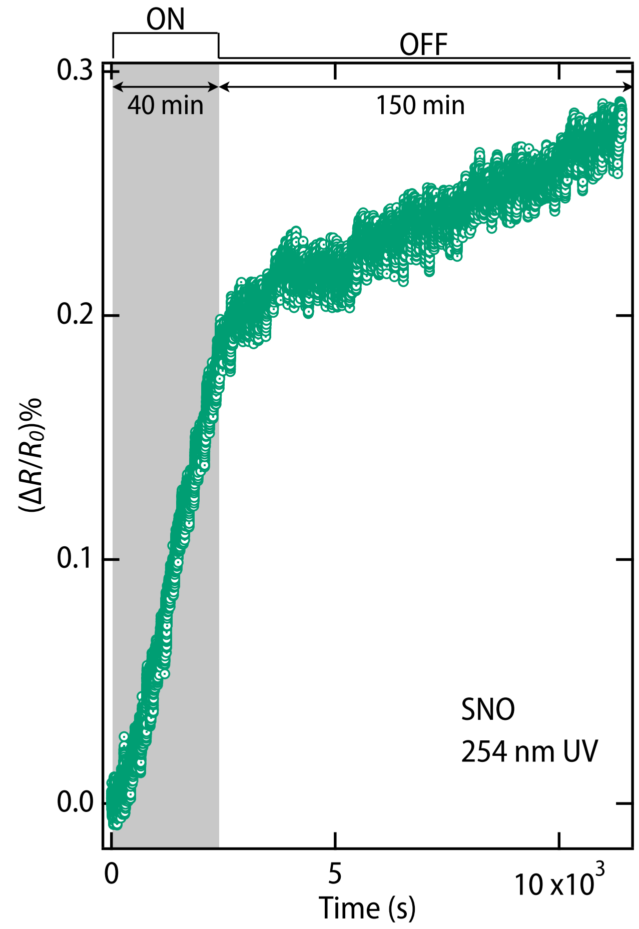

FIG. S7. **Interaction of 254 nm UV light with SNO film at 35°C showing non thermal effect.** RPCR response of the SNO film at 35°C under UV exposure for 40 mins. The RPCR increases with UV illumination, indicating a non-thermal effect, as a temperature rise would otherwise lead to a decrease in RPCR.

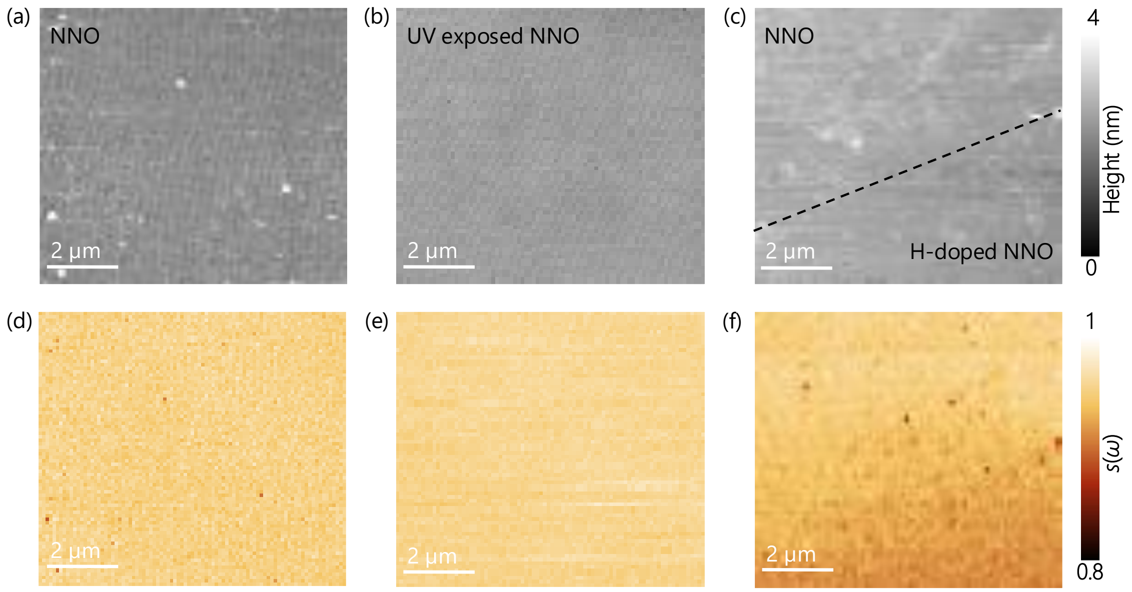

FIG. S8. **Scattering-type scanning near-field optical microscopy of the nickelate films.** (a-c) AFM topography images pristine of NNO, UV exposed NNO, and the interface between pristine and hydrogen-doped NNO, respectively. (d)-(f) Corresponding s-SNOM images, $s(\omega)$ at ω =1000 cm^{-1}. Figures S5(a)–(b) show the atomic force microscopy (AFM) images of pristine NNO and UV-exposed NNO films. Both films exhibit essentially identical surface morphology, with no discernible topographic features that distinguish one from the other. The corresponding near-field images acquired at 1000 cm^{-1} (Figs. S5(d)–(e)) likewise show nearly identical contrast, indicating that both samples have a homogeneous electronic response at the nanoscale. This observation is consistent with electrical transport measurements, which reveal only a small change in resistance in the UV-exposed film. Further, to verify the sensitivity and dynamic range of the s-SNOM technique, we also examined a thin film containing both pristine and hydrogen-doped regions within the same field of view. Figure S5(c) shows the AFM image of the NNO–HNNO interface on a reference sample, revealing no significant morphological differences between the two regions; the dotted line indicates the boundary separating the pristine and H-doped areas. In contrast, the near-field signal from the H-doped NNO is significantly reduced compared to pristine NNO (Fig. S5(f)), consistent with its substantially higher resistivity. The s-SNOM map clearly resolves this electronic discontinuity, capturing the spatial transition from the conductive pristine region to the insulating H-doped region. Together, these AFM and nano-IR measurements demonstrate that variations in near-field contrast arise from differences in local electronic conductivity rather than from surface topography. They also establish that hydrogen doping introduces nanoscale electronic inhomogeneity in NNO, whereas UV exposure does not produce any detectable nanoscale electronic contrast.

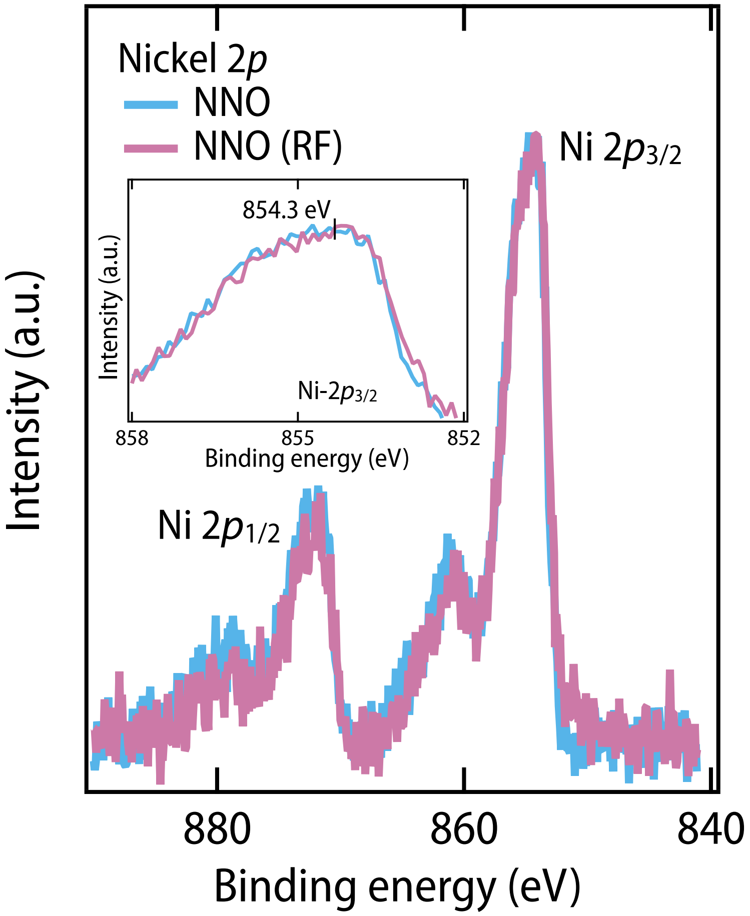

FIG. S9. **XPS at Ni edge of NNO film before and after RF exposure.** Ni-2p XPS spectra of the NNO film after RF exposure, compared with the pristine film, showing unchanged Ni valence. The inset provides an enlarged view of the Ni-2$p_{3/2}$ region.

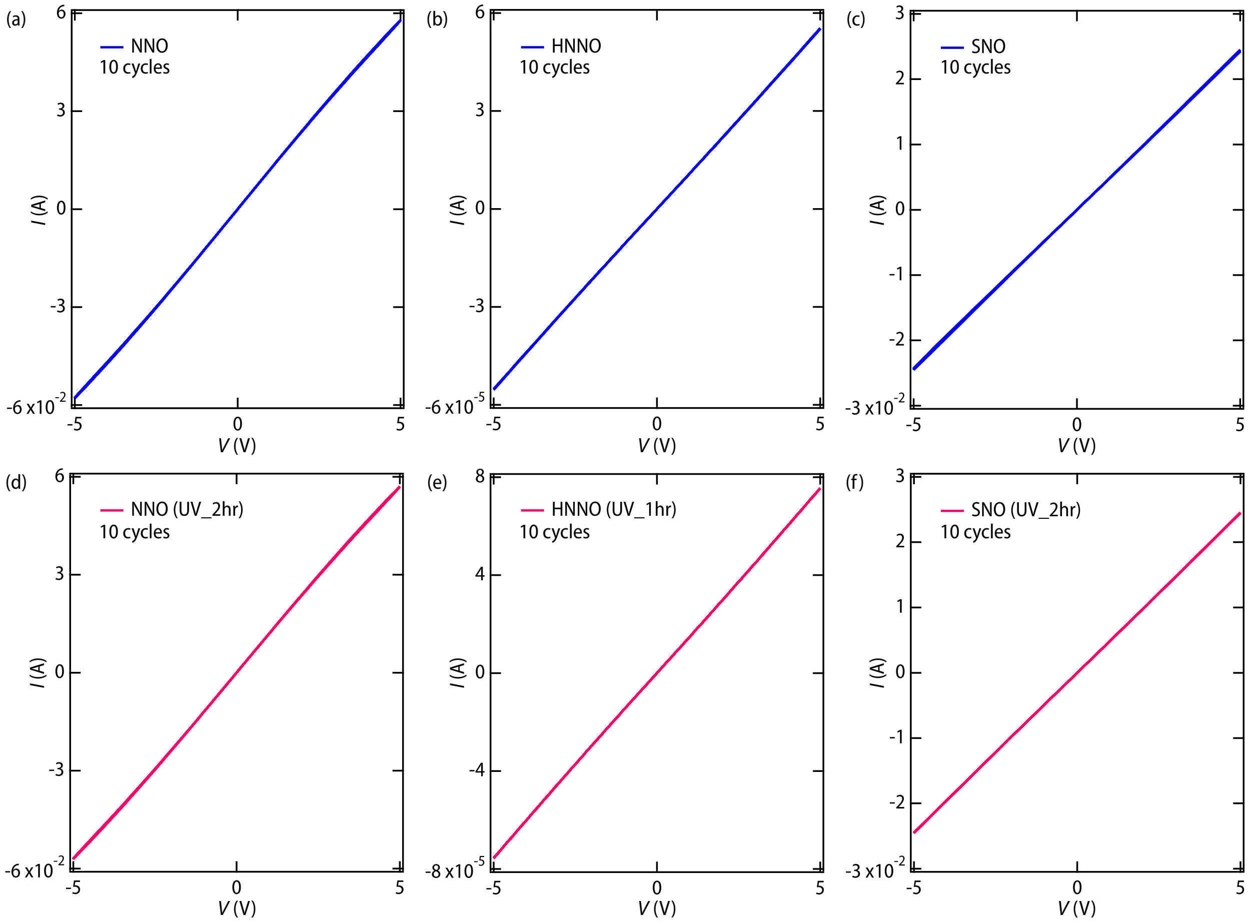

FIG. S10. I-V **characteristics of nickelate films before and after 254 nm UV exposure.** Cyclic I-V measurements of NNO, HNNO, and SNO films measured for 10 consecutive cycles at room temperature (a–c) before and (d–f) after UV exposure, respectively. All devices exhibit linear I-V behavior with negligible hysteresis, confirming ohmic contacts.

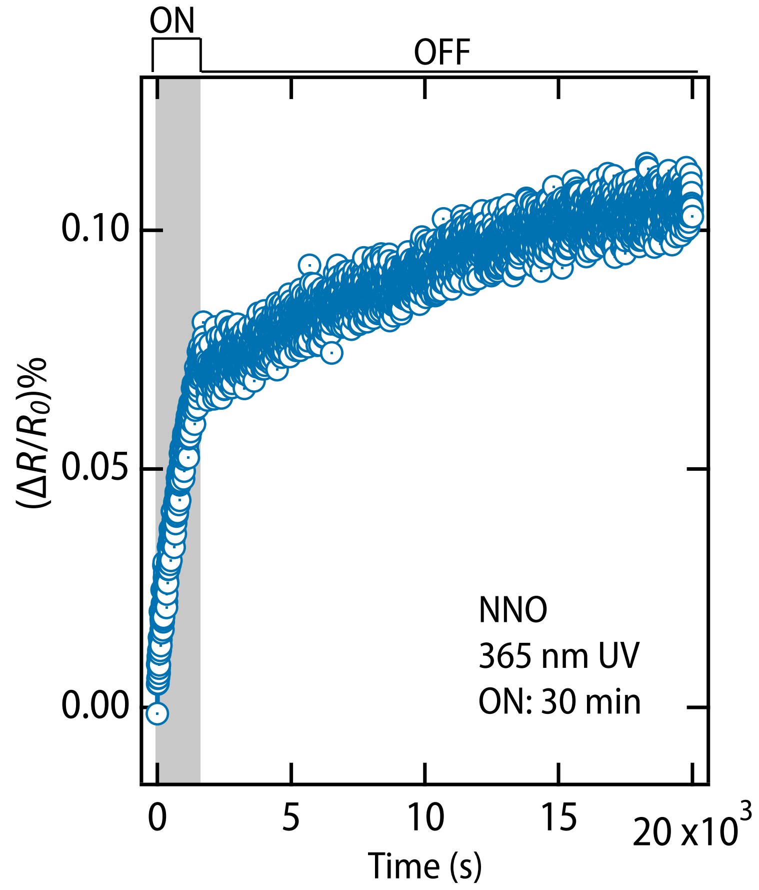

FIG. S11. **Interaction of 365 nm UV light with NNO film at 35°C.** RPCR of the NNO film exposed to UV illumination (365nm) for 30 mins. The RPCR is lower compared to that of a similar exposure with 254 nm UV light.

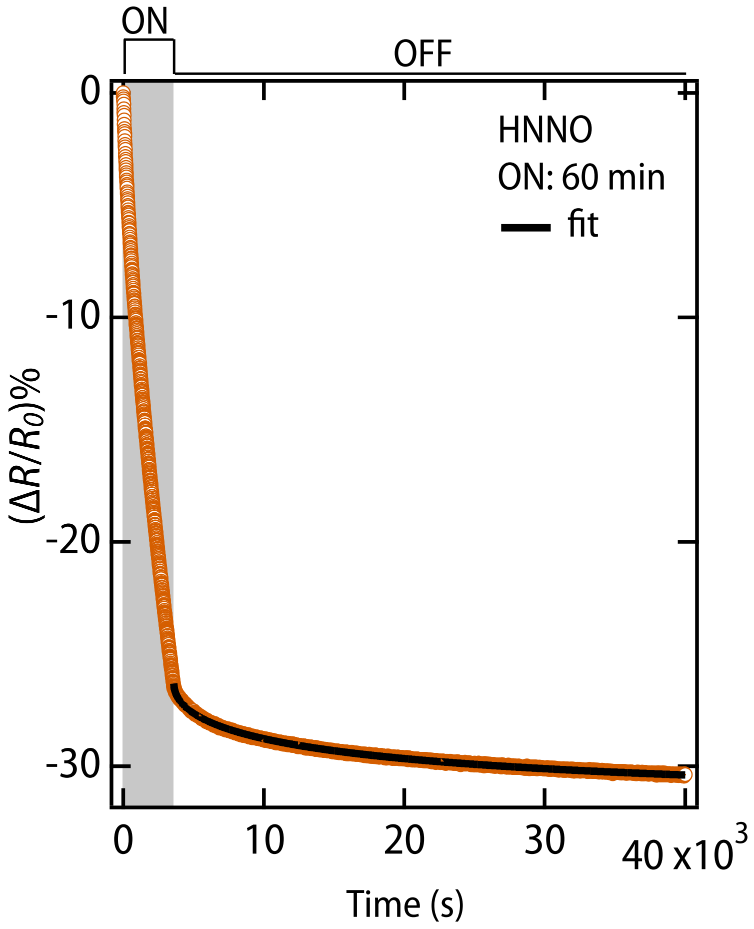

FIG. S12. **Interaction of 254 nm UV light with HNNO film at 35°C.** RPCR of the HNNO film exposed to UV illumination (254 nm) for 60 mins, where the resistance relaxation occurs once the UV light is switched OFF. The black curves depict stretched exponential fits during the light-OFF phase.

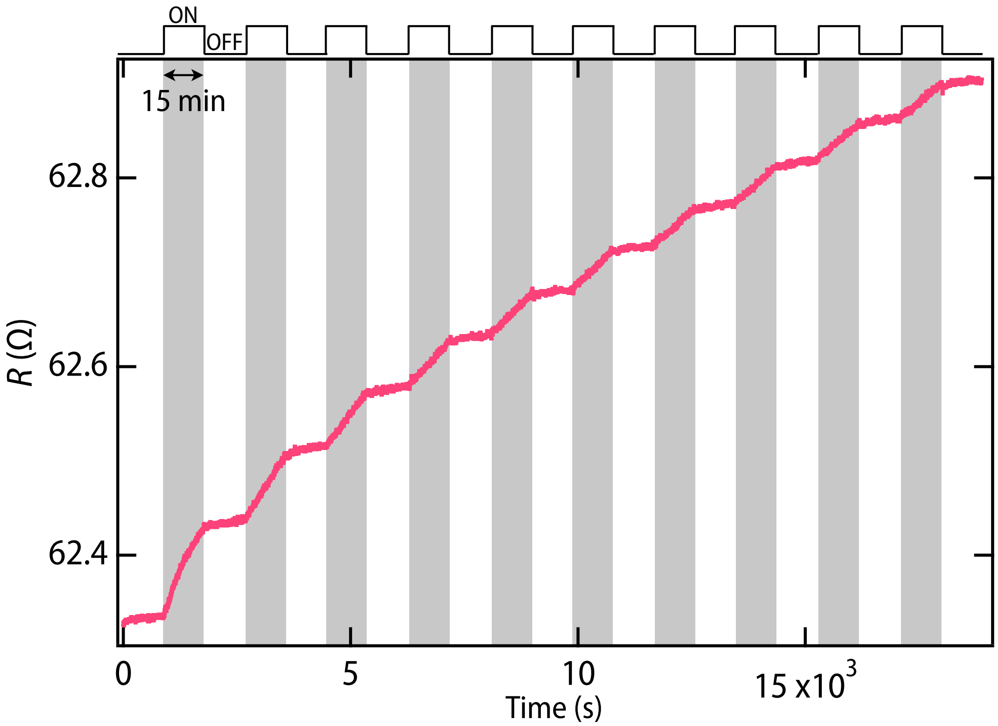

FIG. S13. **Reproducibility test of habituation behavior of NNO film with UV light at 35°C.** (a) Variation in resistance of the NNO film under cyclic exposure to UV light (254 nm), with 15 mins ON and 15 mins OFF intervals, repeated for 10 training cycles.

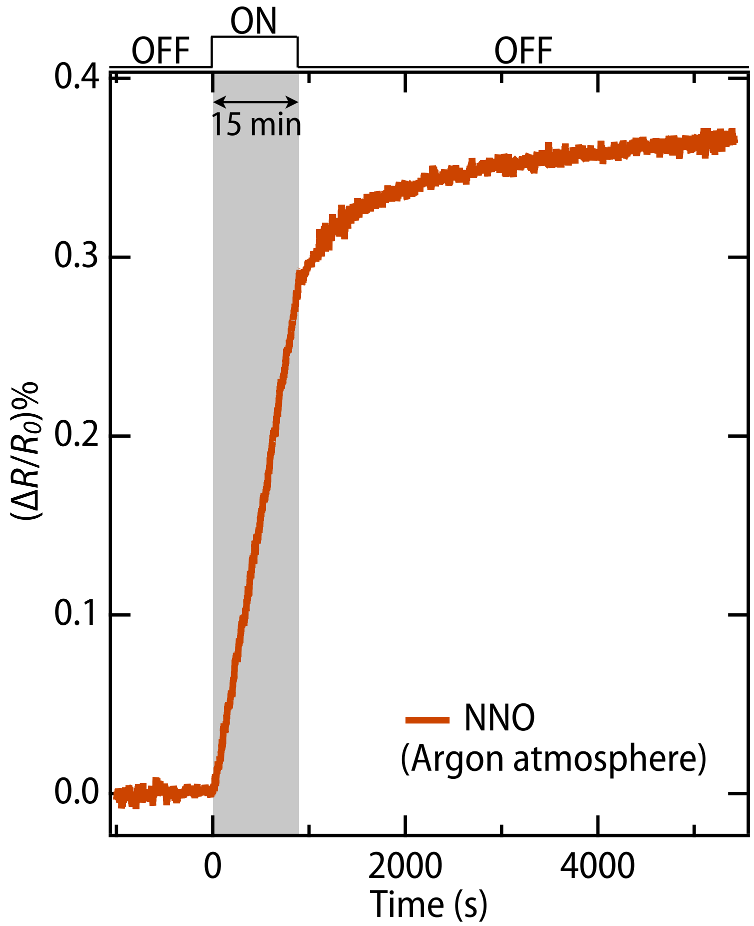

FIG. S14. **Interaction of 254 nm UV light with NNO film in Argon environments at 35°C.** RPCR of the NNO film under 254 nm UV illumination for 15 mins measured in Ar environment.

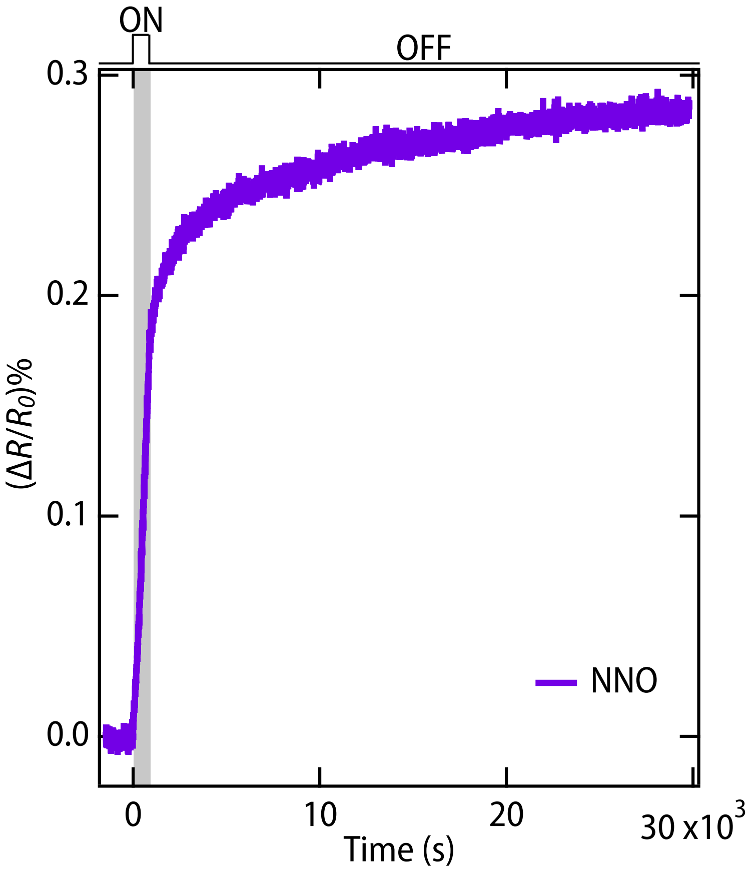

FIG. S15. **Reproducibility test of interaction of 254 nm UV light with NNO film at 35°C.** RPCR of a different NNO film under 254 nm UV illumination for 15 min at 35°C, showing reproducible behavior consistent with that observed in Fig. 5(a) of the main manuscript.

I. MINIMAL DYNAMICAL MODELS FOR UV RESPONSE

In the main text we demonstrated that nickelates show memory-like resistance changes in response to UV light exposure. This is consistent with growing literature that demonstrates similar effects through diverse stimuli, including tensile and compressive strain [1], electric fields [2] or electric fields acting through ionic liquids [3], and electromagnetic radiation, all of which are believed to act through the modulation of oxygen stoichiometry.

Here we consider dynamical systems that capture key qualitative features of the UV response data for the primary nickelate considered in the main text (NNO). In particular, we are interested in minimal models that have as few state variables, parameters, and assumptions as possible, while capturing the qualitative features of the resistance dynamics.

Model for NNO (chiller ON case)

In this section, we study the memory-like response of NNO to UV light (detailed in the main text) from a driven dynamical systems perspective. We seek to model how the resistance $R(t)$ changes in response to a time-dependent stimulus $u(t) = \phi(I(t))$, where $\phi(I) \in [0, 1]$ is a saturating function of the UV light intensity, i.e., $\phi(0) = 0$ and $\lim_{I\to\infty} \phi(I) = 1$. As an example, $\phi(I) = I/(1 + I)$ meets these requirements. Below, we assume $u = 1$ during each "pulse", and $u = 0$ otherwise. The approach is informed by prior work on minimal dynamics for habituation in neurological and single-cell settings [4] as well as non-biological contexts [5].

As shown in Fig. 5 of the main text, $R(t)$ exhibits a pronounced initial rise during continuous UV exposure, and a very slow accumulation phase once the stimulus is removed, which is notably weaker for longer exposure durations (Fig. 5h). The "ON-phase" rise exponentially diminishes with repeated pulses (Fig. 6 of the main text), whereas the "OFF-phase" slow post-exposure rise is well fit by a stretched exponential, suggesting an aging process with a spectrum of timescales likely corresponding to complex changes in oxygen stoichiometry. We approximate this aging process with a minimal set of linear memory variables that cumulatively represent the material's stimulus exposure history. Notably, while a potentially very large number of timescales may be needed to approximate well a stretched exponential, we find that just two memory variables are sufficient to capture qualitative features of the UV-induced resistance dynamics observed during experimental timescales.

Putting the fast ON-phase and the slow OFF-phase rise together, we arrive at the following general model for the response of NNO resistance to UV light:

$$\frac{dm_1}{dt} = \beta_1 u - \alpha_1 m_1 \tag{1a}$$
$$\frac{dm_2}{dt} = \beta_2 u - \alpha_2 m_2 \tag{1b}$$
$$\frac{dR}{dt} = u\gamma_{\text{ON}} + (1-u)\,\gamma_{\text{OFF}} \tag{1c}$$

where the resistance dynamics during UV-ON ($u = 1$) and OFF ($u = 0$) are nonlinear functions of the memory state variables,

$$\gamma_{\text{ON}} = \frac{A}{1+m_1}, \quad \gamma_{\text{OFF}} = \frac{Bm_1}{(1+m_2)^k}. \tag{2}$$

Equation 1a is the prototypical dynamic equation for a leaky memory or leaky storage, which is evident from its analytical solution

$$m_1(t) = m_1(0)\exp(-\alpha_1\, t) + \beta_1 \int_0^t \exp\left(-\alpha_1\left(t-\tau\right)\right) u(\tau) d\tau. \tag{3}$$

The first term in Eq. 3 describes an exponential decay of the initial memory state $m(0)$ with decay rate α_1. The second term describes the discounted-in-time storing of $u(t)$ (the convolution of $u(t)$) with accumulation rate β_1. Taken together, these two terms model a memory that stores information on the exposure to the external signal $u(t)$ with rate β_1 and forgets with rate α_1. The equations for $m_1(t)$ and $m_2(t)$ in Eq. 1 are structurally identical and independent. They serve to model a fast and a slow memory variable, which are used to approximate the response dynamics mentioned above. These fast and slow memory variables may loosely correspond to short-term accumulation and long-term consolidation of oxygen vacancy changes. The effect of accumulation of an external signal on resistance is similar to that in nickelate transistor experiments, where the source to drain resistance changes proportionally to the time-integrated (or "accumulated") gate signal [3]. Similarly, nickelate resistance changes were shown to be proportional to deposited (or "accumulated") energy [2] before.

These functional forms for $\gamma_{\text{ON}}, \gamma_{\text{OFF}}$ belong to the minimal motif class identified in Smart et al. [4], within which qualitative behavior is structurally robust across different functional realizations of the same dynamical ingredients. The rational forms used here are natural representatives of this class. The model is best understood as a local dynamical approximation to the stretched

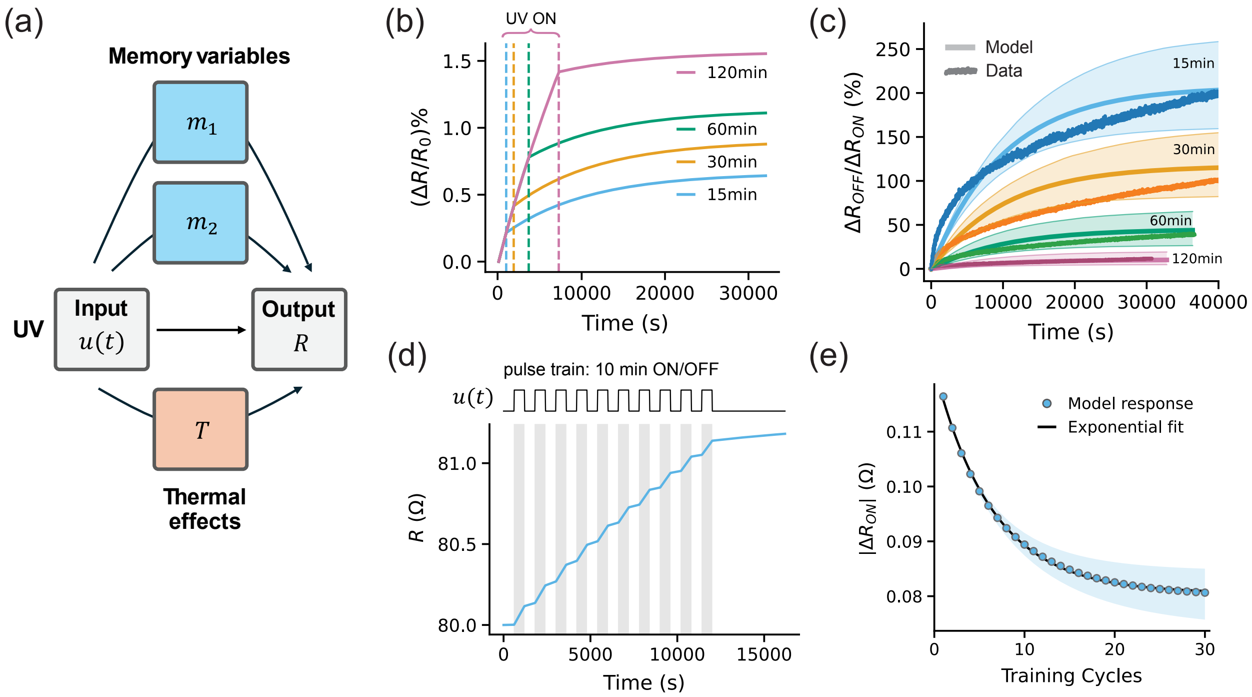

FIG. S16. **Dynamical system modelling of NNO response to UV.** (a) Dynamic UV light exposure $u(t)$ influences material resistance $R(t)$ directly and indirectly through a pool of memory variables m_1, m_2, as well as through temperature T. We simulate Eq. (1) with fixed parameters and variable inputs $u(t)$. (b) Response to single pulses of variable durations. (c) Normalized change in resistance during the UV OFF phase, overlaid with experimental data (dots). Shaded envelopes show the central 80% interval of a Latin Hypercube sensitivity analysis ($N = 50$, $\pm 25\%$ variations in α_1, α_2, k). The correct aging ordering — progressive suppression of the OFF-phase resistance rise with increasing illumination duration — is preserved across the sensitivity envelope, and experimental observations are broadly consistent with the envelope across all four illumination durations. Panels (b) and (c) are normalized as in Figs. 5(a)–(d) and 5(h) of the main text. (d) Model response to a train of pulses. (e) The resistance change per pulse decays exponentially over 30 cycles; shaded envelope as in panel (c). The same parameter set used to capture the aging behavior in panel (c) produces exponential habituation decay without further adjustment. Parameters: $\beta_1 = 1, \alpha_1 = 1, \beta_2 = 2, \alpha_2 = 10^{-2}, A = 2, B = 8, k = 4$. A unit of time corresponds to 10^4 s ≈ 2.8 hr.

exponential phenomenology of the main text on experimentally relevant timescales, rather than a derivation of it. Other functional forms $\gamma_{\mathrm{ON}} = f(m_1, m_2, R), \gamma_{\mathrm{OFF}} = g(m_1, m_2, R)$ are possible– for instance, $\gamma_{\mathrm{OFF}} = \frac{B_1 m_1 + B_2 m_2}{(1+m_2)^k}$ includes contributions from both memory timescales and can better approximate the stretched exponential behavior, at the cost of an additional parameter. It

will be important in future work to connect these functional forms to potential mechanisms for UV-induced material changes such as oxygen vacancy kinetics and slow consolidation [2].

Figure S16a presents a schematic showing how UV light influences resistance through both memory variables and temperature. We find that this family of models can capture key qualitative features of the responses in the main text. In particular, Figures S16(b)-(c) demonstrate that the proposed model successfully recapitulates the qualitative behavior of the NNO film's UV responses shown in Figs. 5(a)–(d) and 5(h) of the main text, respectively. Additionally, Figs. S16(d)-(e) capture the response to repeated stimuli and the habituation behavior presented in Figs. 6(b)–(c) of the main text. Equation 1 thus provides an initial model of memory-driven resistance dynamics in a controlled temperature setting. Below, we briefly outline how this model can be extended to the case when the chiller is OFF.

Incorporating thermal effects

The previous model captures memory-driven resistance dynamics under the chiller ON conditions, where temperature effects are actively suppressed. We now extend this framework to the chiller "OFF" case, where UV illumination leads to stimulus-induced heating, and temperature changes significantly influence the resistance.

In the chiller OFF setting, both UV exposure and material heating contribute to the resistance response. The temperature $T(t)$ rises as a result of UV exposure and relaxes back toward ambient room temperature T_{room} when the stimulus is paused. To account for this, we augment the original model by adding a dynamical equation for temperature and a temperature-dependent term in the resistance evolution:

$$\begin{aligned}\frac{dm_i}{dt} &= \beta_i u - \alpha_i m_i \\ \frac{dR}{dt} &= u\gamma_{\text{ON}} + (1-u)\,\gamma_{\text{OFF}} + \kappa\,(R - R(T)) \\ \frac{dT}{dt} &= \beta_T u - \eta(T - T_{\text{room}}).\end{aligned} \tag{4}$$

The resistance response to temperature shifts is governed by a prescribed function $R(T)$ (as in Figs. 2b, c of the main text). The parameter κ controls the rate at which resistance relaxes toward the temperature-dependent target $R(T)$, β_T sets the rate of UV-induced heating, and η governs the rate of thermal dissipation back to ambient temperature T_{room}.

The light-induced resistance dynamics (γ_{ON} and γ_{OFF}) may now also depend on temperature, and the accumulation and decay rates of the memory variables may likewise be temperature-dependent.

This thermal extension is motivated by the known sensitivity of nickelate resistance to temperature changes (see Fig. 2 of the main text for NNO). In the absence of active cooling, UV illumination increases the material temperature, which independently alters resistance. Including $T(t)$ in the model allows for a unified description of memory effects and thermal effects, and will be important for interpreting data in uncontrolled temperature conditions (e.g., Fig. S16a).

Finally, we note that thermal dissipation is rapid when the chiller is active. This corresponds to the limit $\eta \to \infty$ which restrains $T(t) \approx T_{\mathrm{room}}$. In this limit, the system simplifies to Eq. 1 above, where temperature dynamics can be neglected.

---

[1] F. Conchon, A. Boulle, R. Guinebretière, E. Dooryhée, J.-L. Hodeau, C. Girardot, S. Pignard, J. Kreisel, and F. Weiss, Journal of Physics: Condensed Matter **20**, 145216 (2008).

[2] M. Kotiuga, Z. Zhang, J. Li, F. Rodolakis, H. Zhou, R. Sutarto, F. He, Q. Wang, Y. Sun, Y. Wang, N. A. Aghamiri, S. B. Hancock, L. P. Rokhinson, D. P. Landau, Y. Abate, J. W. Freeland, R. Comin, S. Ramanathan, and K. M. Rabe, Proceedings of the National Academy of Sciences of the United States of America **116** (2019), 10.1073/pnas.1910490116.

[3] J. Shi, S. D. Ha, Y. Zhou, F. Schoofs, and S. Ramanathan, Nature Communications **4** (2013), 10.1038/ncomms3676.

[4] M. Smart, S. Y. Shvartsman, and M. Mönnigmann, Proceedings of the National Academy of Sciences **121**, e2409330121 (2024), https://www.pnas.org/doi/pdf/10.1073/pnas.2409330121.

[5] M. Smart, S. Y. Shvartsman, and M. Mönnigmann, in *2024 IEEE 63rd Conference on Decision and Control (CDC)* (2024) pp. 577–582.
